# Polymeric frustrated Lewis pairs in CO_2_/cyclic ether coupling catalysis†

**DOI:** 10.1039/d2sc00894g

**Published:** 2022-03-08

**Authors:** Thomas A. R. Horton, Meng Wang, Michael P. Shaver

**Affiliations:** Department of Materials, School of Natural Sciences, The University of Manchester Manchester UK michael.shaver@manchester.ac.uk; Sustainable Materials Innovation Hub, Royce Hub Building, The University of Manchester Oxford Road Manchester UK

## Abstract

Frustrated Lewis pairs (FLPs) are now ubiquitous as metal-free catalysts in an array of different chemical transformations. In this paper we show that this reactivity can be transferred to a polymeric system, offering advantageous opportunities at the interface between catalysis and stimuli-responsive materials. Formation of cyclic carbonates from cyclic ethers using CO_2_ as a C_1_ feedstock continues to be dominated by metal-based systems. When paired with a suitable nucleophile, discrete aryl or alkyl boranes have shown significant promise as metal-free Lewis acidic alternatives, although catalyst reuse remains illusive. Herein, we leverage the reactivity of FLPs in a polymeric system to promote CO_2_/cyclic ether coupling catalysis that can be tuned for the desired epoxide or oxetane substrate. Moreover, these macromolecular FLPs can be reused across multiple reaction cycles, further increasing their appeal over analogous small molecule systems.

## Abstract 2

Polymeric frustrated Lewis pairs catalyse the coupling of epoxides and oxetanes with CO_2_ with high selectivity under mild CO_2_ pressures across multiple reaction cycles.

## Graphical abstract

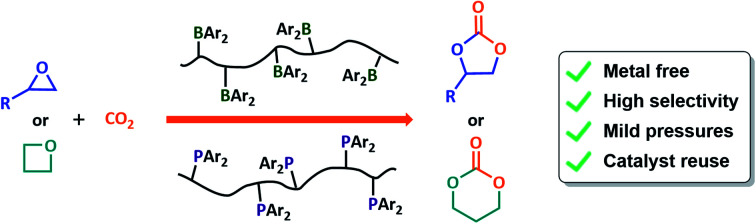

## Introduction

Valorisation of CO_2_ as a renewable carbon feedstock is desirable in the pursuit of a sustainable, carbon-neutral society. Current efforts to use this typically unreactive substrate rely either on designer gas-capture systems and subsequent reactions, or combining CO_2_ directly with suitably reactive substrates.^1^ Epoxides are one such reactive substrate that has been extensively explored, where insertion of CO_2_ yields the corresponding cyclic or polycarbonate products.^2^ While the majority of early research efforts in this area have employed expensive or non-abundant metal-based systems, the use of metal-free catalysts to facilitate these transformations has expanded in recent years.^3^

Frustrated Lewis pairs (FLPs) are ubiquitous as metal-free catalysts for a myriad of chemical transformations.^4–7^ The frustration induced by sterically hindered Lewis acid (LA) and base (LB) centres permits cooperative action of, and catalysis with, a multitude of substrates including carbon dioxide, dihydrogen and cyclic ethers.^8–15^ In 2017, our group demonstrated that FLP reactivity is maintained when incorporated into a polymeric system, revealing a new class of stimuli-responsive materials that exploit FLP-mediated small molecule activation.^16,17^ Inspired by these systems, Yan *et al.* later reported similar macromolecular FLPs capable of activating CO_2_ and catalysing amine formylation.^18^ Polymeric FLPs have also been reported in C–H functionalisation, amination and hydrogenation catalysis with demonstrated potential for catalyst recovery and reuse.^19–22^

**Fig. 1:**
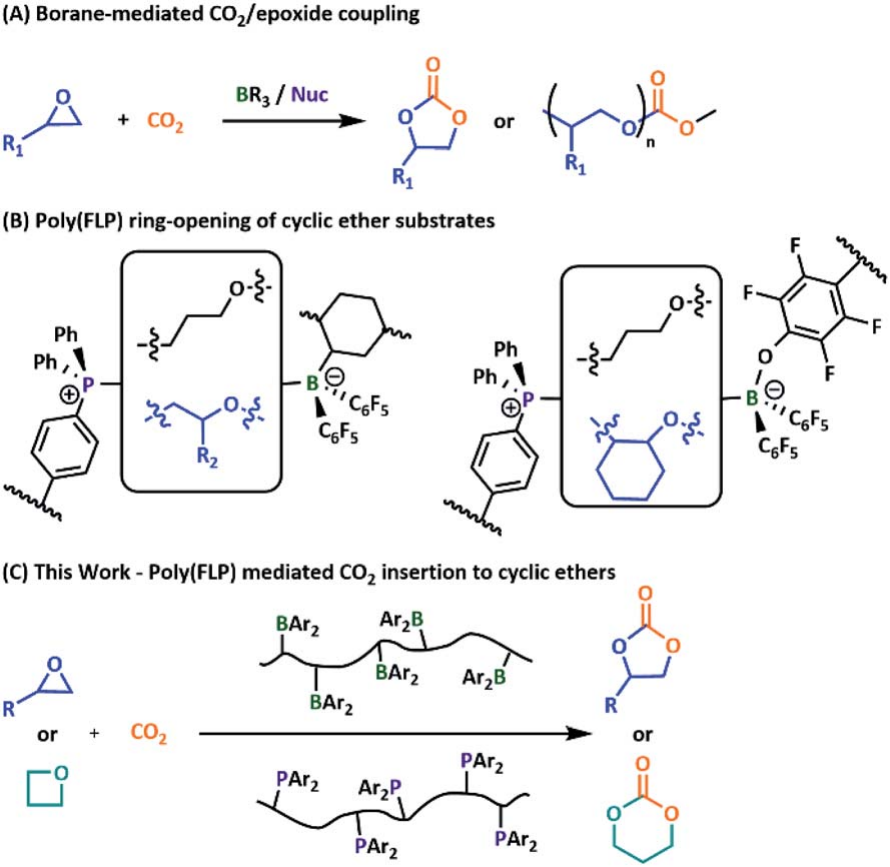
(A) Previously reported borane-mediated CO_2_ insertion catalysts. (B) Cyclic ether triggered poly(FLP) networks. (C) Poly(FLP) catalysed cyclic carbonate formation.

Recently, we reported the successful crosslinking of highly Lewis acidic styrenic copolymers with a corresponding Lewis basic copolymer, *via* ring-opening of cyclic ether substrates.^23,24^ Given these results, we wondered whether our systems would enable effective catalytic insertion of CO_2_ into the ring-opened cyclic ether substrates. Aryl and alkyl boranes have previously been applied successfully in the formation of both cyclic and polycarbonates when paired with a phosphonium or ammonium salt partner.^25–31^ More recently, superbasic phosphazenes have also proved active under mild conditions.^32^ The use of a phosphine LB in these reactions is however, to our knowledge, previously unreported. Herein, we report the first use of both conventional small molecule and polymeric FLPs to catalyse the insertion of CO_2_ into cyclic ether substrates with high selectivity towards the cyclic product (Fig. 1).

## Results and discussion

### Screening of LA/LB copolymer catalysts

**Fig. 2:**
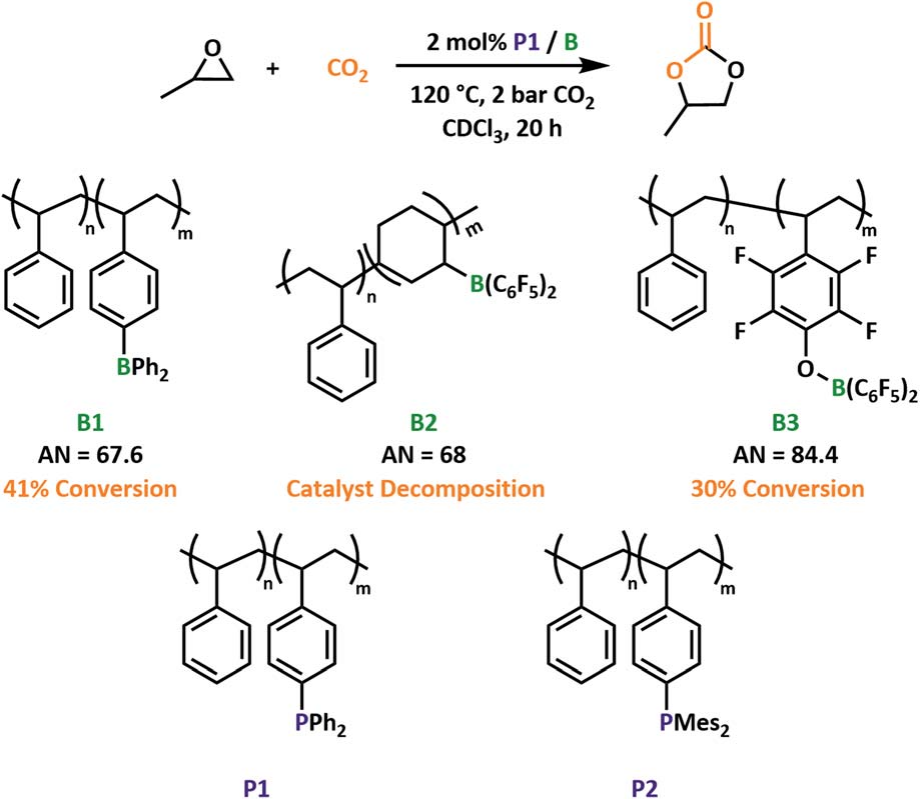
Performance of polymeric LAs for CO_2_ insertion to PO when paired with P1. Structure of P2 to be used in later reaction screening. AN = acceptor number calculated using the Gutmann–Beckett method.

Having previously reported three boron-containing copolymers of varying Lewis acidity (B1–3) we first sought to optimise the LA component of our proposed system (Fig. 2). Propylene oxide (PO) was selected as a model substrate. A readily synthesised triphenylphosphine functionalised copolymer, P1, was selected as the Lewis basic co-catalyst. For the PO/P1/B2 system, conversion to propylene carbonate was observed but remained low, as the catalysis promoted reverse hydroboration to decouple the catalytic site from the polymer backbone (Fig. S5 and S6†). We know that the addition of epoxides affords stable networks, suggesting that the addition of CO_2_ promotes decomposition after the first turnover. Decomposition occurs at both moderate and elevated temperatures and mimics reversible reactions commonly associated with polymeric alkyl boranes,^33^ therefore preventing further catalytic testing using B2.

Aryl-boron-containing polymers have a higher thermal stability.^21^ This was proven when pairing a polymeric LB with B1 and B3. While the capacity of B3 to ring-open cyclic ethers was already established,^24^ we anticipated B1 would also facilitate this reaction based on previous small-molecule systems.^15,27^ Previous studies into small molecule borane catalysed formation of cyclic carbonates reported a reactivity decrease when using stronger LAs as a result of adduct stability.^27^ Indeed, the same trend can be observed within our polymeric systems when considering estimations of Lewis acidity by the Gutmann–Beckett method.^34,35^ Reactions employing B3 (acceptor number, AN = 84.4) were notably slower than those with the less Lewis acidic B1 (AN = 67.6) under the same optimised conditions (Fig. 2 and S5†). Given its superior performance, we selected B1 as the Lewis acidic component for the rest of this study.

With catalytic conversions using B1 remaining low, optimisation of the system was essential. We hypothesised that catalyst decomposition was occurring, as extended reaction times did not increase product formation. Analysis of the reaction mixture by ^31^P NMR spectroscopy revealed the presence of a new oxidised phosphine species, a commonly encountered off-cycle product during phosphine-promoted catalysis.^36^ As direct CO_2_ activation is not observed with this FLP system under the mild conditions used in catalysis, the mechanism likely proceeds *via* ring-opening of the epoxide substrate, followed by CO_2_ insertion as reported with other aryl borane-containing systems.^27^ Triphenylphosphine oxidation was not observed in the absence of epoxide, meaning that an on-cycle decomposition process involving PO must be occurring. Deoxygenation could potentially form propene, however analysis of the crude reaction mixture by ^1^H NMR spectroscopy revealed no obvious side products, potentially due to their gaseous nature.

As we previously noted that ring-opening of styrene oxide (SO) by poly(FLP) systems was rapid,^23^ we hoped to use this substrate to better understand this catalytic system. Under the same conditions, only 19% conversion from SO to styrene carbonate was observed. However, the system showed increased phosphine oxidation and the concomitant formation of styrene as confirmed by the presence of vinyl peaks at 5.3 ppm and 5.8 ppm in ^1^H NMR spectra (Fig. S7 and S9†). Inspired by this finding, we re-attempted the reaction with PO under milder conditions and preventing gaseous product release, and indeed observed propene *in situ* (Fig. S8†).

**Fig. 3:**
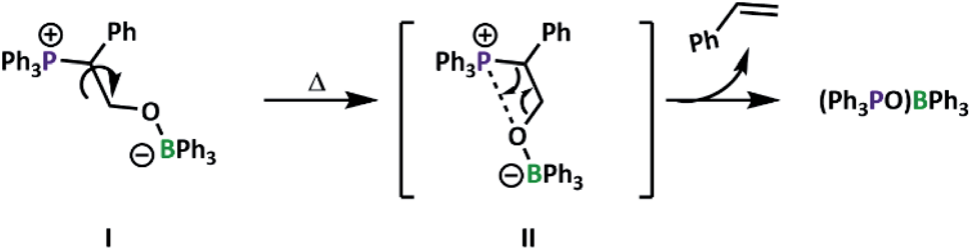
Proposed route of catalyst deactivation for P1, represented using small-molecule model compounds.

Evidence of alkene formation prompted us to consider the possible routes to epoxide deoxygenation that would result in phosphine oxidation. Previous work had shown ring-opening of epoxides by tertiary phosphines results in Wittig-like reactivity,^37^ while heating an FLP activated N_2_O complex releases N_2_ gas, forming a P=O–B linked species.^38^ Ring-opening of episulfides, the sulfuric analogue of epoxides, also leads to formation of similar linkages.^15^ It is thus hypothesised that, at elevated temperatures, CO_2_ insertion is competitive with alkene elimination and phosphine oxidation, gradually leading to catalyst decomposition (Fig. 3).

Catalyst activity could thus be renewed through the addition of disiloxane reducing agents^36,39–42^ used to regenerate the phosphine. Indeed, we found that addition of 1,3-diphenyldisiloxane (DPDS) to our PO/B1/P1/CO_2_ reaction mixture allowed for >99% conversion of PO (Fig. S10†). Although DPDS addition increased catalytic performance, the mix of desired carbonate products and alkene side products obtained when using SO both rendered the reaction less green and made product separation more difficult.

**Fig. 4:**
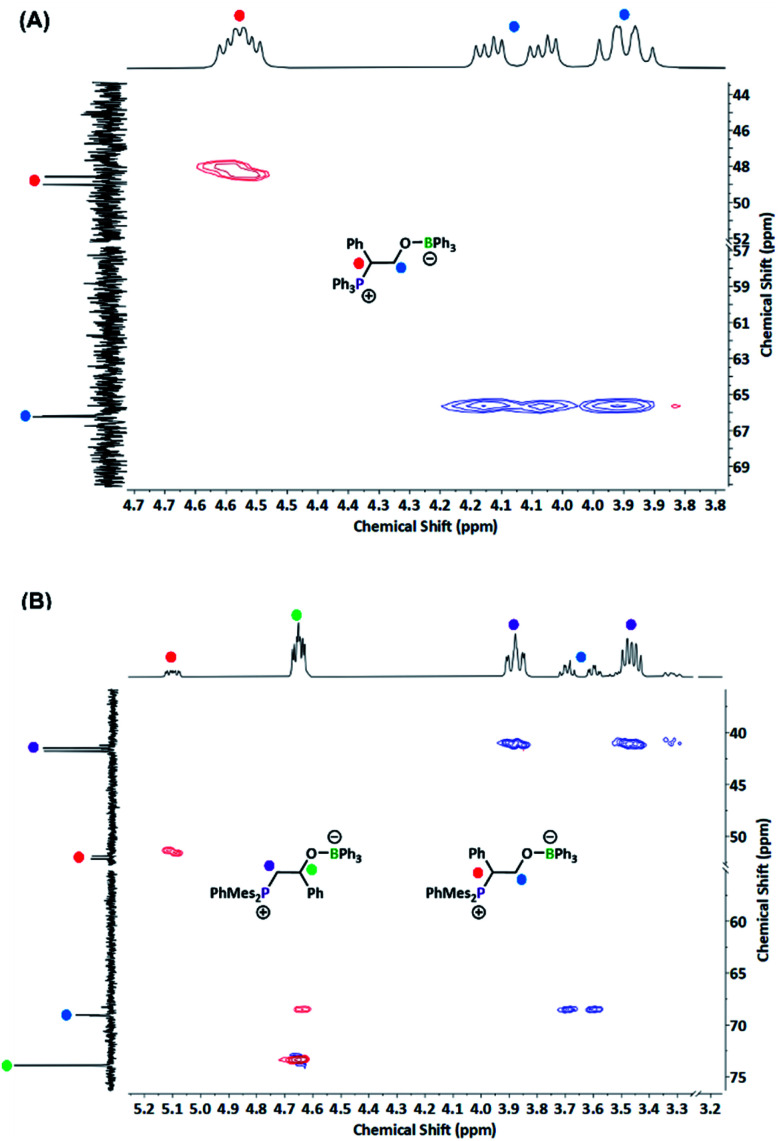
HSQC analysis demonstrating the influence of phosphine steric bulk on mode of SO ring-opening. Red = CH/CH_3_, blue = CH_2_. (A) P1/B1 system, attack of phosphine LB observed exclusively at more hindered carbon. (B) P2/B1 catalytic system, attack of phosphine LB at least hindered carbon is preferred by a factor of 7. Solvent peaks omitted for clarity.

From this understanding, we sought to design a system to preclude this decomposition pathway. In N_2_O systems, elimination of N_2_ occurred when the phosphorus and boron components were oriented in the less energetically favourable *cis* conformation.^38^ If the same conformational requirements are present, as previous small molecule studies suggested the *cis* configuration is preferred for PO activation by a BPh_3_/P*t*Bu_2_Me FLP pair,^15^ a bulkier polyphosphine may be preferred, especially as PPh_3_ has a lower cone angle than P*t*Bu_2_Me.^43^ This doesn't preclude decomposition from the *trans* configurations as attack of the phosphine copolymer on SO occurs at the more hindered carbon of the epoxide ring (*i.e.* adjacent to the phenyl ring) as assigned by ^31^P–^13^C coupling constants (44.5 Hz, Fig. 4A). With this mode of attack, rotation from conformations I to II (Fig. 3) affords the undesired conformation, with decomposition encouraged by the enthalpic driving force of conjugated product formation (styrene).

The switch to our previously reported mesityl-substitute P2 (ref. 16) would increase both steric bulk (favouring the *trans* attack and precluding rotation) and increase Lewis basicity to enhance its reactivity. The impact of this increased steric bulk from the mesityl groups was readily observable using small molecule model compounds, with a 7 : 1 preference for ring-opening at the less hindered carbon observed for SO (Fig. 4B). Under the same reaction conditions, the P2/B1 catalyst combination led to >99% conversion of SO to styrene carbonate. Notably, no vinyl peaks were observed in the reaction mixture by ^1^H NMR spectroscopy (Fig. S13†), with minimal phosphine oxidation observed by ^31^P NMR spectroscopy. Taken together, the mesityl groups and polymer backbone bulk mitigate unproductive alkene elimination.

### Substrate scope

With an optimised system in hand, we investigated the substrate scope and overall reactivity of the P2/B1 system. Conversion of PO to propylene carbonate reaches >99% conversion in just 7 hours, dramatically outperforming the less Lewis basic P1. Realising that CO_2_/epoxide catalysis with small molecule FLPs is also unreported, we compared our macromolecular catalysts to their small molecule equivalents, BPh_3_ and PMes_2_Ph. These conventional FLPs, under the same reaction conditions, gave >99% conversion of PO within just 6 hours (Fig. S27†), the first example of conventional FLP use in CO_2_ insertion to epoxides. Use of a B/P system therefore significantly outperforms previously reported organocatalytic systems which typically require higher catalyst loadings.^3,44^ Use of a phosphine LB also allows for milder conditions than ammonium salt systems,^29^ while maintaining the same selectivity, although phosphonium systems are substantially more active.^27^

**Fig. 5:**
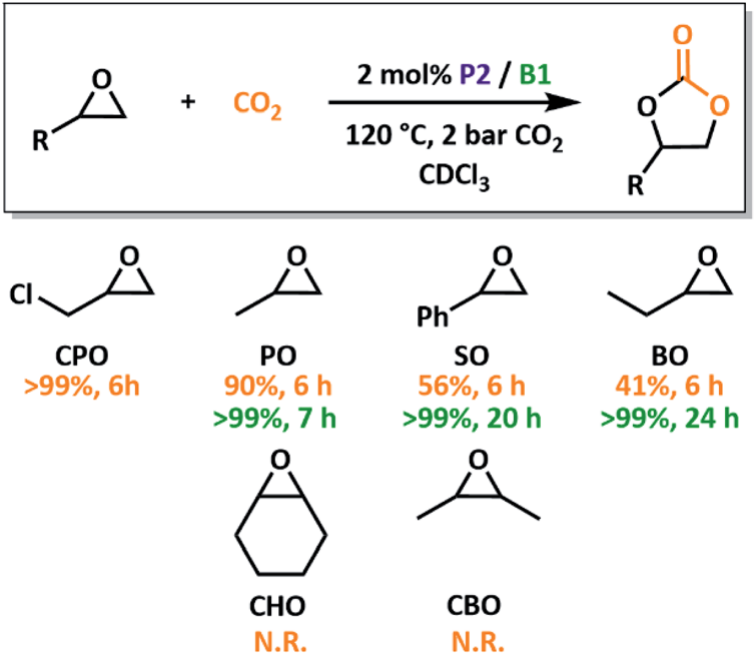
Screening of cyclic ether substrates for CO_2_ insertion to obtain cyclic carbonate products. Conversions calculated by ^1^H NMR spectroscopy after 6 h are shown in orange (N.R. = no reaction) and the times required for >99% conversion are shown in green. Reactions were carried out in a sealed ampoule on a 0.8 mmol scale using 2 bar CO_2_ pressure and catalyst P2/B1 (2 mol% as calculated from the number of expected active functional groups).

A variety of substrates were screened for P2/B1 using similar reaction conditions (Fig. 5). All terminal epoxide substrates were quantitatively converted in under 24 hours, although their rates differed markedly depending on sterics and electronics. 3-Chloropropylene oxide (CPO) outperformed all other tested substrates, consistent with trends reported for other systems.^27,45^ While the electron withdrawing chlorine promotes rapid turnover, the sterically similar methyl group (butylene oxide, BO) shows decreased conversion. The role of steric bulk was also apparent for internal epoxides. While ring-opening of cyclohexene oxide (CHO) is possible using both P2/B1 and our previously reported poly(FLP) systems,^23,24^ productive conversion into product is not observed. Previous attempts to use conventional FLPs in the copolymerisation of CHO and carbonyl sulfide were also unsuccessful,^26^ although borane/phosphonium salts do form polycarbonates.^27,28^ To check if this was due to the strain induced during CO_2_ insertion preventing cyclohexyl rearrangements, *cis*-2,3-butylene oxide, CBO, was trialled. With no conversion for this derivative, it is likely down to steric clashes during ring-closure that prevent product formation.

### Reactivity with oxetane substrates

Notably, oxetane (OX) can also be successfully ring-opened by P2/B1. Conversion to trimethylene carbonate (TMC) was slower due to the lower ring strain of OX relative to epoxide substrates,^46,47^ as well as the relatively high strength of the intermediate crosslinked networks formed as reported in our previous work.^23^ Attempts to expand the OX substrate scope with both 3-bromooxetane and 3,3-dimethyloxetane were unsuccessful. Although a change in chemical shift was observed in ^11^B NMR spectra, indicating LA coordination, no change was observed by ^31^P NMR spectroscopy for the LB counterpart, suggesting steric clashes prevent nucleophilic attack. Selectivity for cyclic 6-membered carbonates over their polymeric counterparts is often cited as problematic when using a CO_2_ insertion synthetic route.^48^ With epoxide substrates, high selectivity for the cyclic product is achieved at all temperatures screened. However, if the high temperatures optimised for epoxide ring opening are used (120 °C) with oxetane, only 61% preference for the cyclic product TMC (Table 1, entry 1) is observed. Lowering the temperature to 100 °C gives a significant improvement in selectivity, although longer reaction times are required to achieve high conversions (Table 1, entry 2 and 3). This additionally supports the formation of the polycarbonate product directly, rather than *via*TMC polymerisation. Interestingly, while better leaving groups have been proposed as routes to improve cyclic product selectivity in small molecule catalysts,^29^ the opposite trend is observed herein where the better P1 leaving group gave less selectivity (Table 1, entry 4).

**Table 1:** Effect of temperature and LB on oxetane/CO_2_ coupling conversion and selectivity

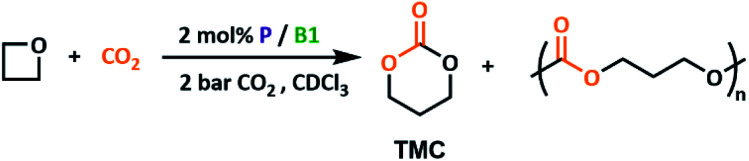					
Entry	LB	*T* (°C)	Time (h)	Con.^a^ (%)	% TMC^a^
1	P2	120	24	57	61
2	P2	100	24	32	76
3	P2	100	72	83	81
4	P1	120	24	26	31

a Conversion determined using ^1^H NMR spectroscopy (ESI Fig. S31–S34).

### Catalyst recyclability

**Fig. 6:**
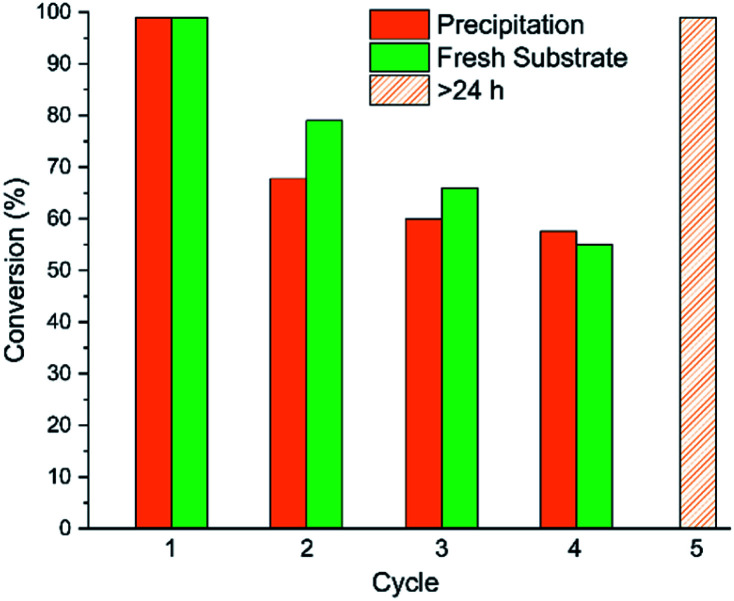
Conversions obtained in consecutive reaction cycles for CO_2_ insertion to PO, as calculated by ^1^H NMR spectroscopy, using 1,3,5-trimethoxybenzene as an internal standard. Reaction conditions as stated in Fig. 5. The data shown in green was obtained by adding fresh substrate at the end of each cycle (*i.e.* without purification and catalyst recovery). The recovered P2/B1 catalysts remain active after 4 cycles, achieving >99% conversion with extended reaction times (cycle 5).

Polymer-supported catalysts have also shown potential for facile reuse or incorporation into flow reactors as an enabler for green manufacturing.^49,50^ Previous polymeric Lewis bases have been synthesised in pursuit of multiple use cycles but require more forcing conditions and suffer from limited substrate scopes.^51,52^ We thus decided to explore the catalytic performance of P2/B1 across multiple reaction cycles (Fig. 6) using a precipitation-recovery strategy (Section 6, ESI†), comparing it to the spiking of the reaction mixture with fresh substrate. This more rigorous recovery procedure is a true stress test of catalyst reuse using P2/B1.

As can be seen in Fig. 6, and for either reuse methodology, catalytic performance decreases significantly for P2/B1 after the first reaction cycle. Smaller rate decreases are observed over subsequent cycles. Gradual phosphine oxidation can be observed (Fig. S40†), although attempted regeneration using DPDS did not substantially increase reaction rates, as the additional steric bulk from the mesityl groups could prohibit silane attack.^39^ It is important to note that this is a reduction in rate only, as extending the reaction time gives quantitative conversion (Fig. 6, cycle 5 and S39†).

It is important to highlight that these macromolecular catalysts are generated *in situ*, with the addition of substrate triggering gelation. This means that the accessibility of the catalyst active site is a key factor in reuse efficiency. Post-reaction, we observed that the catalyst resting state was present in its gelated, cross-linked form, supported by ^31^P and ^11^B NMR spectroscopy, and GPC measurements of hydrodynamic volume (Fig. S43 and S44†). Precipitation thus forms a superstructure that does not dissolve, but instead swells (Fig. S42†), creating a physical constraint on substrate access to catalytic sites.^20^ In our case, crosslinking occurs at the active components, limiting polymer chain segmental motions in the vicinity of the FLP and likely suppressing the rate of epoxide capture and CO_2_ insertion. This hypothesis is consistent with the observation that a significant decrease in activity is only observed after the first cycle. Future work in the team directed at incorporating these metal-free catalysts into flow reactors is an important step in overcoming these practical constraints.

## Conclusions

In summary, we report the first use of both conventional and polymeric FLPs in the formation of carbonates from cyclic ethers using CO_2_, with high selectivity for the cyclic carbonate product. The synthesised macromolecular catalysts displayed good reactivity for a variety of terminal epoxide substrates and are also active catalysts for the formation of trimethylene carbonate from oxetane and CO_2_. Post-reaction, the poly(FLPs) used can be easily recovered and reused, with gradual decreases in catalyst efficiency attributed to partial phosphine oxidation as well as increased crosslinking. Further tuning of both the catalytic system and reaction conditions is expected to offer a more diverse substrate scope with higher selectivity for oxetane substrates. Further development of intermolecular poly(FLPs) for catalysis is expected to instead utilise the effects seen from crosslinking to enhance reactivity with other substrates *via* compartmentalisation.

## Author contributions

Thomas A. R. Horton and Meng Wang: investigation, methodology and manuscript writing. Michael P. Shaver: conceptualisation, manuscript writing – review and editing, supervision and funding acquisition.

## Conflicts of interest

There are no conflicts to declare.

## Supplementary Material

SC-013-D2SC00894G-s001
