# Supplementary material for: Polymeric frustrated Lewis pairs in CO2/cyclic ether coupling catalysis
Source: Chem Sci. 2022 Mar 8;13(13):3845–50. doi: 10.1039/d2sc00894g (PMC8966658; doi:10.1039/d2sc00894g)
Supplement: SC-013-D2SC00894G-s001 [file SC-013-D2SC00894G-s001.pdf]

Electronic Supplementary Material (ESI) for Chemical Science.
This journal is © The Royal Society of Chemistry 2022

Supporting Information for

Polymeric Frustrated Lewis Pairs in CO_2/Cyclic Ether Coupling Catalysis

Thomas A. R. Horton, Meng Wang and Michael P. Shaver*

Department of Materials, School of Natural Sciences, The University of Manchester, Manchester, UK.

Sustainable Materials Innovation Hub, Royce Hub Institute, The University of Manchester, Oxford Road, Manchester, UK.

*michael.shaver@manchester.ac.uk

Table of Contents

1. Materials, Reagents and Methods 2
   General Considerations 2
   Materials 2
   Instrumentation 2
2. Polymer Synthesis and Characterisation 3
   General procedure for synthesis of poly(styrene-*co*-4-styryl-phosphines), P1 and P2 via anionic polymerisation 3
      Characterisation of poly(styrene-co-4-styryl-diphenylphosphine), P1: 3
      Characterisation of poly(styrene-*co*-4-styryl-dimesitylphosphine), P2: 4
   Synthesis of poly(styrene-*co*-4-styryl-diphenylborane), B1 5
   Summary of polymerisation data 6
3. Catalytic Screening for Epoxide Substrates 7
   General procedure for CO_2 insertion to cyclic ether substrates: 7
   Poly(Lewis Acid) screening: 8
   Poly(Lewis Base) screening 10
      Screening of CO_2 insertion to epoxides using P1/B1 10
      Screening of CO_2 insertion to epoxides using P2/B1 14
4. Mechanistic Investigations 19
   P1 small molecule model systems 19
      Activation of propylene oxide, PO: 19
      Activation of styrene oxide, SO 24

P2 Small molecule model systems 28
Activation of propylene oxide, PO 28
Activation of styrene oxide, SO 32
CO_2 Activation and Proposed Mechanism 36
5. CO_2 Insertion to Oxetane Substrates 37
6. Consecutive Reaction Cycles 40
General reaction procedure 40
7. References 44

1. Materials, Reagents and Methods

General Considerations

All reactions requiring inert conditions, and their associated manipulations/purifications, were carried out under a nitrogen atmosphere using either a glovebox or standard Schlenk techniques (equipped with a drying column containing copper catalyst and 4Å molecular sieves) unless otherwise stated. All reactants, reagents and solvents used in the reported procedures were dried prior to their use. All glassware used in O_2 and moisture sensitive reactions was dried in an oven at 200 °C for a minimum of 12 hours before use.

Materials

Anhydrous dichloromethane (DCM), diethyl ether, *n*-hexane, pentane, tetrahydrofuran (THF) and toluene were obtained from an MBraun 7 Solvent Purification System containing alumina and copper catalysts under argon gas. Each solvent was degassed via 3 successive freeze-pump-thaw cycles prior to their use. Deuterated solvents (Sigma-Aldrich, Flurochem) were stirred over calcium hydride (Acros Organics) overnight and distilled under an inert atmosphere/reduced pressure before being stored over 3Å molecular sieves. 3-bromo oxetane, 1-butylene oxide (**BO**), 3-3dimethyl oxetane, propylene oxide (**PO**), styrene, styrene oxide (**SO**) and trimethylene oxide (**OX**), all purchased from Sigma-Aldrich, 3-chloropropylene oxide (**CPO**, Fluorochem) and *cis*-2,3-butylene oxide (Acros Organics) were all stirred over calcium hydride overnight before distillation under an inert atmosphere prior to use. 2-bromomesitylene (Aldrich), 4-chlorostyrene (Fluorochem), triisopropyl borate (Fluorochem) and triphenylphosphine (Sigma-Aldrich) were used as received. Magnesium turnings (Sigma-Aldrich) were dried in an oven at 200 °C for a minimum of 3 days prior to their use in Grignard reactions. Carbon dioxide (N5.0, research grade, 99.999% purity) was purchased from BOC.

4-styryl-diphenylborane, 4-styryl-diphenylphosphine, 4-styryl-dimesitylphosphine and 4-phenyl-dimesitylphosphine were synthesised according to previous reports by our group.[1,2] Triphenylborane was synthesised using a route inspired by the reported synthesis of 4-styryl-diphenylborane. 1,3-diphenyldisiloxane (DPDS) was synthesised using a variation on a previously reported synthesis.[3]

Instrumentation

^{1}H, ^{11}B, ^{13}C, ^{31}P and all 2D nuclear magnetic resonance (NMR) data were obtained using either a 400 MHz Bruker AVIII (BBFO 5 mm probe) or 400 MHz Bruker AVIII HD (BBO 5 mm probe) spectrometer. All air and/or moisture sensitive samples were analysed in Young's tap NMR tubes. Molecular weights of polymer samples were determined using an Agilent 1260 Infinity II Multi-Detector Gel Permeation Chromatography (GPC) system

through PLgel 5 µm columns packed with PSDVB beads. Samples were run in THF at a flow rate of 1 mL min^{-1} at 35 °C. Molecular weights were subsequently determined using triple detection based on a calibration obtained using narrow dispersity polystyrene standards.

2. Polymer Synthesis and Characterisation

Polymers **B2** and **B3** were synthesised according to our previous reports and their polymerisation data summarised in table S1.[4,5]

General procedure for synthesis of poly(styrene-*co*-4-styryl-phosphines), P1 and P2 via anionic polymerisation

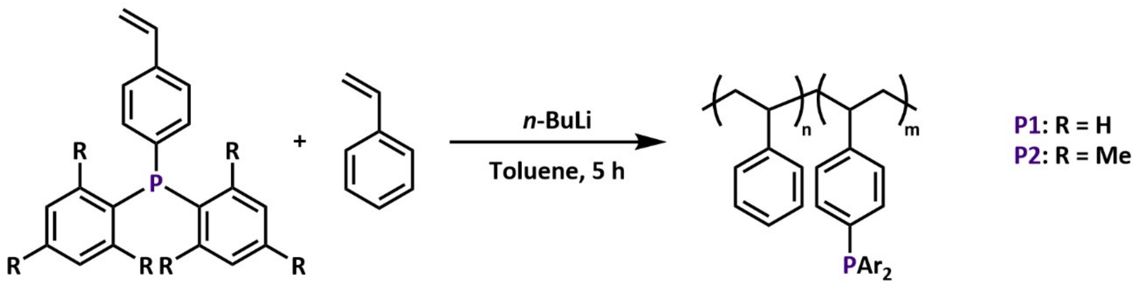

In a glovebox, pre-weighed amounts of styrene (4.78 g, 45.9 mmol) and selected 4-styryl phosphine monomer (2.42 mmol) were dissolved in toluene (15 mL) and stirred to ensure efficient mixing. A solution of *n*-BuLi (1.6M in hexane, 0.16 mL, 0.26 mmol) was added to initiate the polymerisation, turning the reaction mixture a dark red. After stirring for 5 hours, the reaction vessel was removed from the glovebox and a small amount of degassed methanol added to terminate the polymerisation. The colourless mixture was subsequently precipitated twice into methanol, before filtration and drying under reduced pressure gave the desired product as a fine white powder.

Characterisation of poly(styrene-co-4-styryl-diphenylphosphine), P1:

M_n / g mol^{-1} 21000 (theoretical), 22300 (experimental), Đ = 1.2

^{1}H NMR (400 MHz, 295 K, Tol-d_8) δ / ppm 7.22 – 6.71, 6.61 – 6.01 (br, aryl), 2.00 – 1.54 (m, br, alkyl), 1.48 - 1.01 (br, alkyl)

^{31}P NMR (162 MHz, 295 K, Tol-d_8) δ / ppm -6.27 (br)

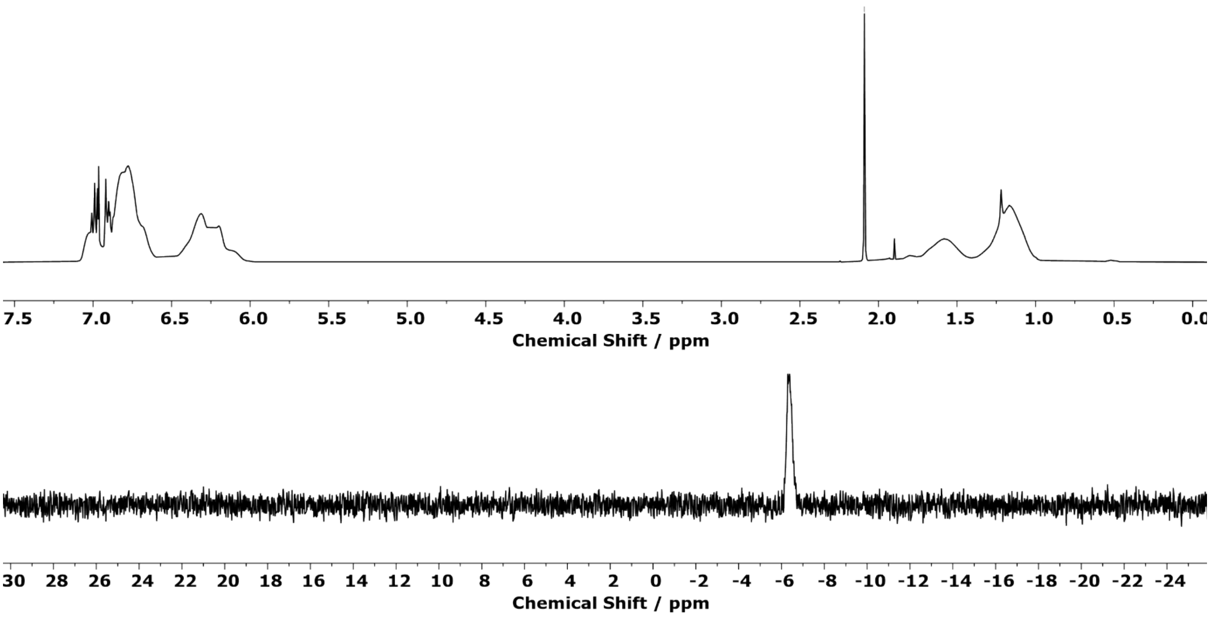

Figure S1: ^{1}H (top) and ^{31}P (bottom) NMR spectra of poly(styrene-*co*¬4-styryl-diphenylphosphine), **P1**.

Characterisation of poly(styrene-*co*-4-styryl-dimesitylphosphine), P2:

M_n / g mol^{-1} 21800 (theoretical), 24700 (experimental), Đ = 1.1

^{1}H NMR (400 MHz, 295 K, Tol-d_8) δ / ppm 7.21 – 6.87 (br, aryl), 6.83 – 6.29 (m,br, aryl), 2.31 – 1.67 (m, br, alkyl), 1.61 - 1.23 (br, alkyl)

^{31}P{^{1}H} NMR (162 MHz, 295 K, Tol-d_8) δ / ppm -22.9 (br)

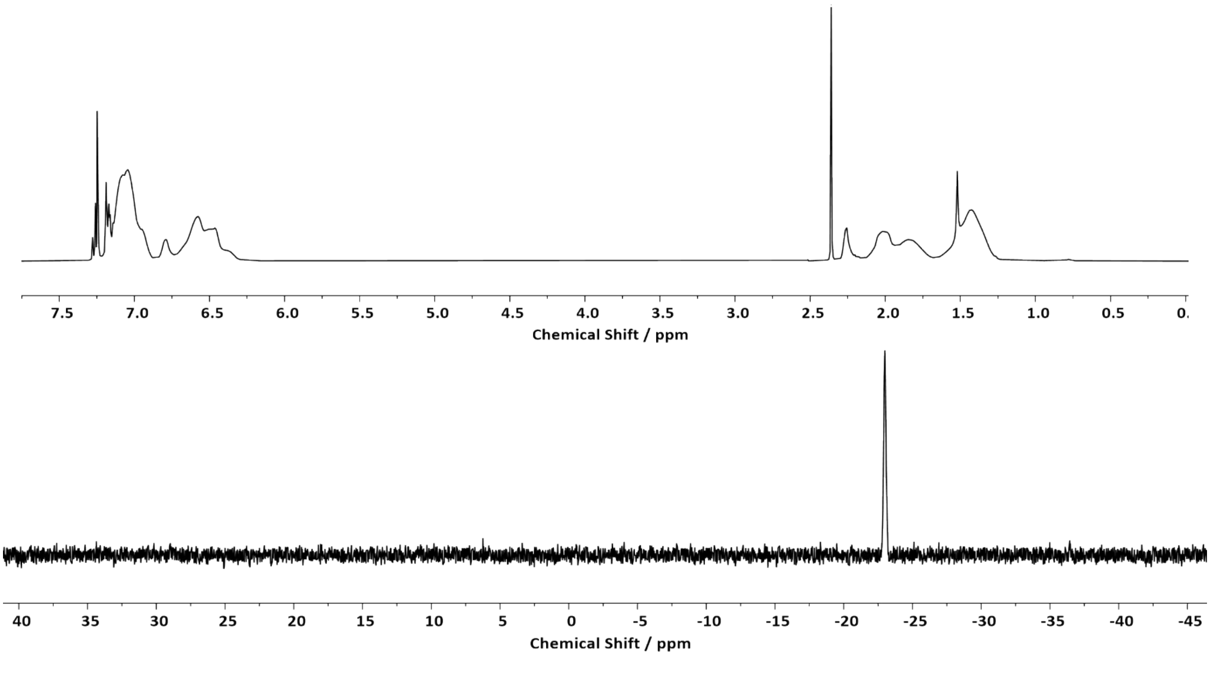

Figure S2: ^{1}H (top) and ^{31}P (bottom) NMR spectra of poly(styrene-*co*-4-styryldimesitylphosphine) **P2**.

Synthesis of poly(styrene-*co*-4-styryl-diphenylborane), B1

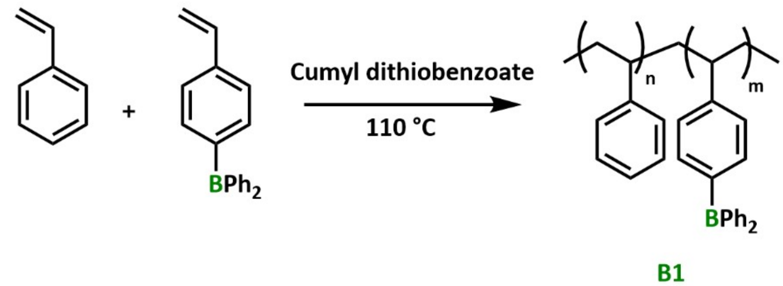

B1

In a glovebox, pre-weighed amounts of styrene (2.58 g, 24.7 mmol), 4-styryl-diphenylborane (0.346 g, 1.29 mmol) and cumyl dithiobenzoate (0.022 g, 0.0829 mmol) were combined in an ampoule. A small amount of 1,3,5-triisopropyl trioxane (TITO) was subsequently added and an aliquot of solution taken for analysis by ^{1}H NMR spectroscopy. The ampoule was then removed from the glovebox and heated to 110 °C for 17 hours, promoting polymerisation. After the desired time, the resultant viscous liquid was diluted with a small amount of toluene and a small aliquot was collected for analysis by NMR spectroscopy. The mixture was then precipitated three times into anhydrous hexane, before filtration and drying under vacuum gave the desired copolymer product as a pink powder (0.9 g, 58% yield).

M_n / g mol^{-1} 18700 (theoretical), 23200 (experimental), Đ = 1.2

^{1}H NMR (400 MHz, 295K, Tol-d_8) δ / ppm 7.63 – 7.24 (m, br, aryl), 7.11 – 6.72 (br, aryl), 6/68 – 6.21 (br, m, aryl), 2.01 – 1.06 (m, br, alkyl)

^{11}B NMR (128 MHz, 295K, Tol-d_8) δ / ppm not observed (n.o.)

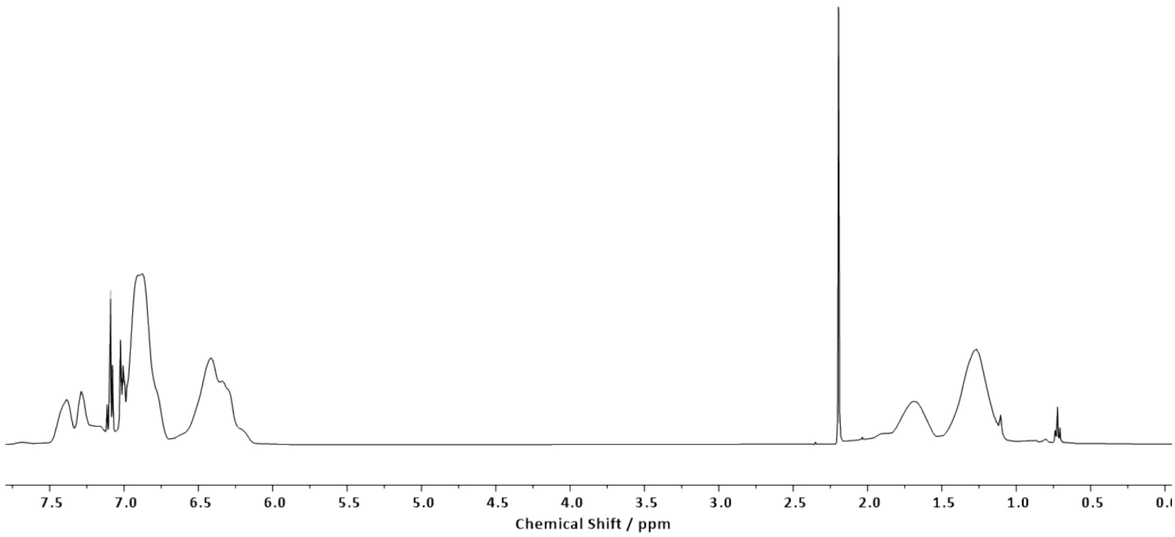

Figure S3: ^{1}H NMR spectrum of poly(styrene-*co*-4-styryl-diphenylborane), **B1**.

Summary of polymerisation data

Table S1: Summary of polymerisation data and example of mol% calculation for catalysis.

	Feed Ratio		Average units per chain (theoretical)		M_n theoretical	M_n GPC	Đ
	Styrene	Functional Monomer	Styrene	Functional Monomer			
**P1**	95	5	181	9[a]	21000	23200	1.2
**P2**	95	5	181	9[a]	21800	24700	1.1
**B1**	95	5	147	14[b]	18700	23200	1.2
**B2**	90	10	165	18	25000	32000	1.2
**B3**	90	10	42	10	6200	7700	1.3

[a] In the absence of unpolymerized monomer for **P1** and **P2** in crude NMR spectra, full conversion was assumed. Theoretical average units were thus calculated on the basis of molar feed ratios.

[b] Calculated considering conversions found in crude NMR spectra relative to internal standard.

All mol% values reported for catalytic reactions utilised the theoretical average units of functional monomer. Reactions were carried out on a 0.8 mmol scale, therefore using 0.016 mmol of each polymer catalyst – P/B. Using **P1** this gives:

0.016 ÷ 9 = 1.8E µmol polymer.

$$\left(\frac{1.8}{1 \times 10^6}\right) \times 21000 = 0.038 \text{ g polymer per reaction.}$$

3. Catalytic Screening for Epoxide Substrates

General procedure for CO_2 insertion to cyclic ether substrates:

In a glovebox, two separate stock solutions of **P polymer** (0.2 mL, 1.8 µmol P moieties) and **B polymer** (0.2 mL, 1.8 µmol B moieties) in $CDCl_3$ were combined in an ampoule and stirred to ensure efficient mixing before addition of a 1,3,5-trimethoxybenzene internal standard solution (0.08 g in 0.1 mL $CDCl_3$). Next, a solution of the desired cyclic ether substrate (0.8 mmol, in 0.2 mL $CDCl_3$) was added and a small portion taken and dilute for t_0 analysis by ^{1}H NMR spectroscopy. The reaction vessel was then sealed with a Young's tap and removed from the glovebox before being connected to a Schlenk line equipped with a CO_2 cylinder via a 3-way joint connection. The reaction mixture was degassed via 3 successive freeze-pump-thaw cycles before being allowed to warm to room temperature. Maintaining an inert atmosphere with stirring, CO_2 was added (2 bar) before sealing of the ampoule and placing in an oil bath at 120 °C for the desired reaction time.

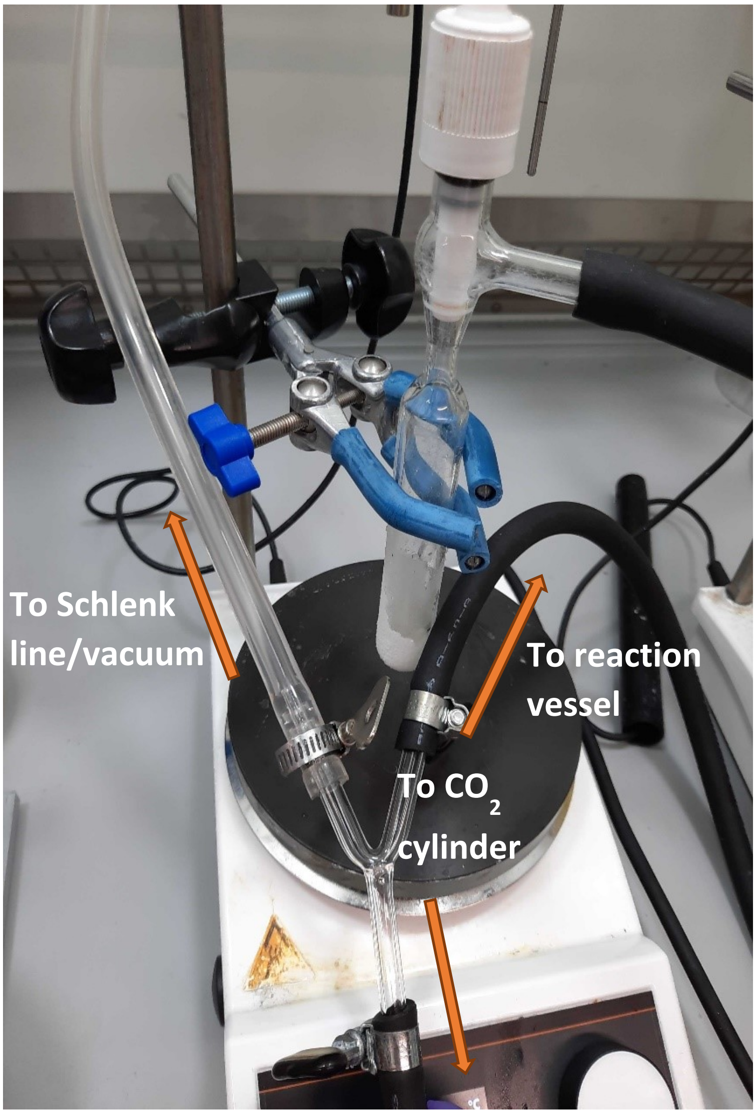

Figure S4: Reaction setup for CO_2 loading to reaction vessel containing catalyst and cyclic ether substrate in $CDCl_3$.

Poly(Lewis Acid) screening:

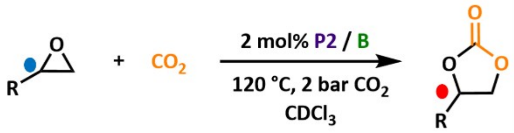

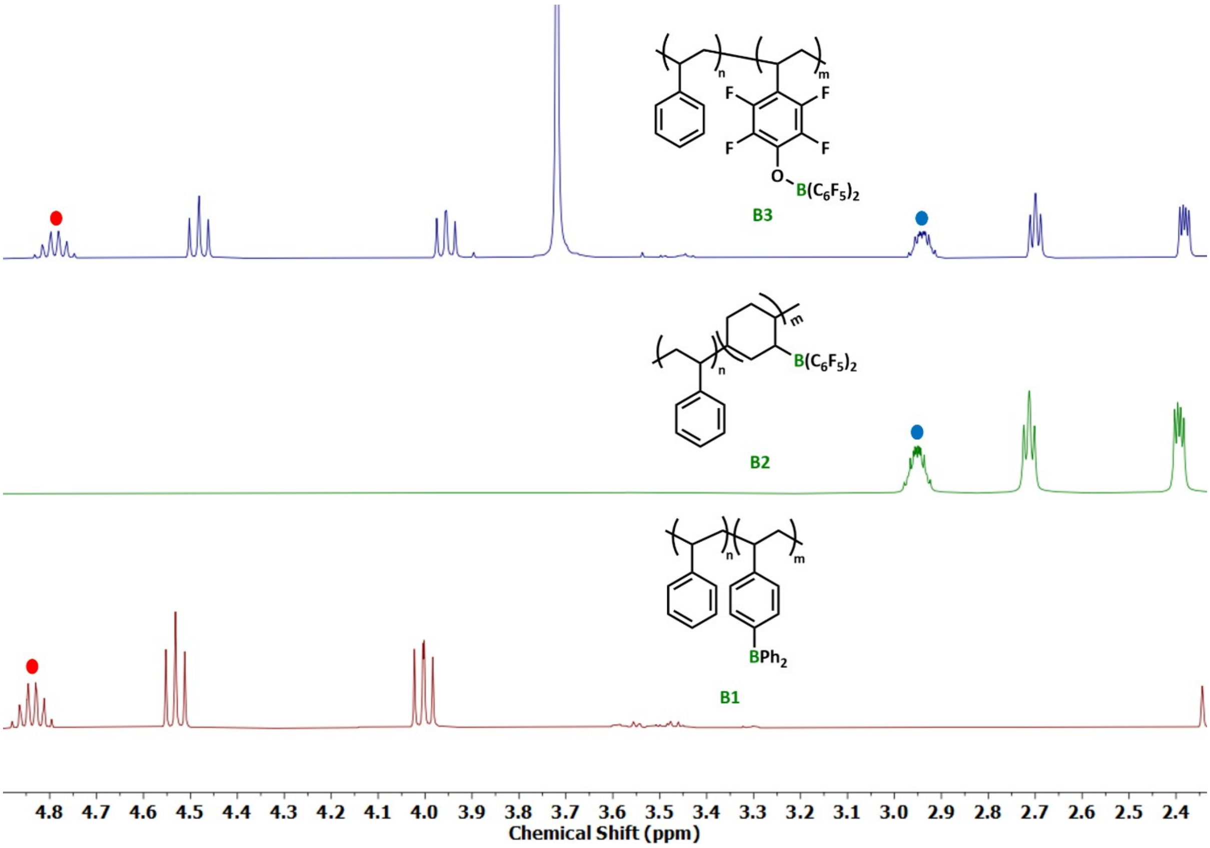

Figure S5: LA polymer screening using **PO** as a substrate with **P2**. Higher Lewis acidity leads to slowed reactivity (**B3**, top vs **B1**, bottom) while polymeric alkyl boranes are prone to decomposition (**B2**, middle).

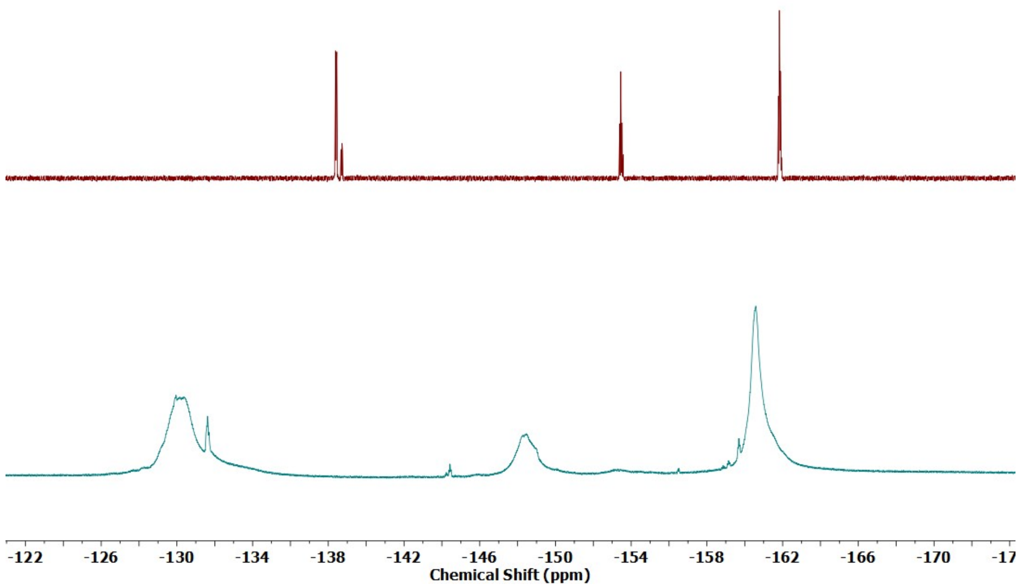

Figure S6: ^{19}F NMR spectra showing decomposition of **B2** (bottom) during catalysis (top), signal sharpening and shift due to reverse hydroboration.

Poly(Lewis Base) screening

Screening of CO_2 insertion to epoxides using P1/B1

Lower than anticipated conversions were obtained when using **P1** as the poly(LB) component. Additional vinyl peaks can be observed via ^1H NMR spectroscopy both when using styrene oxide (**SO**) as a substrate, and when studying the reaction of propylene oxide (**PO**) in situ under milder reaction conditions. An oxidised phosphine peak can also be observed via ^{31}P NMR spectroscopic studies.

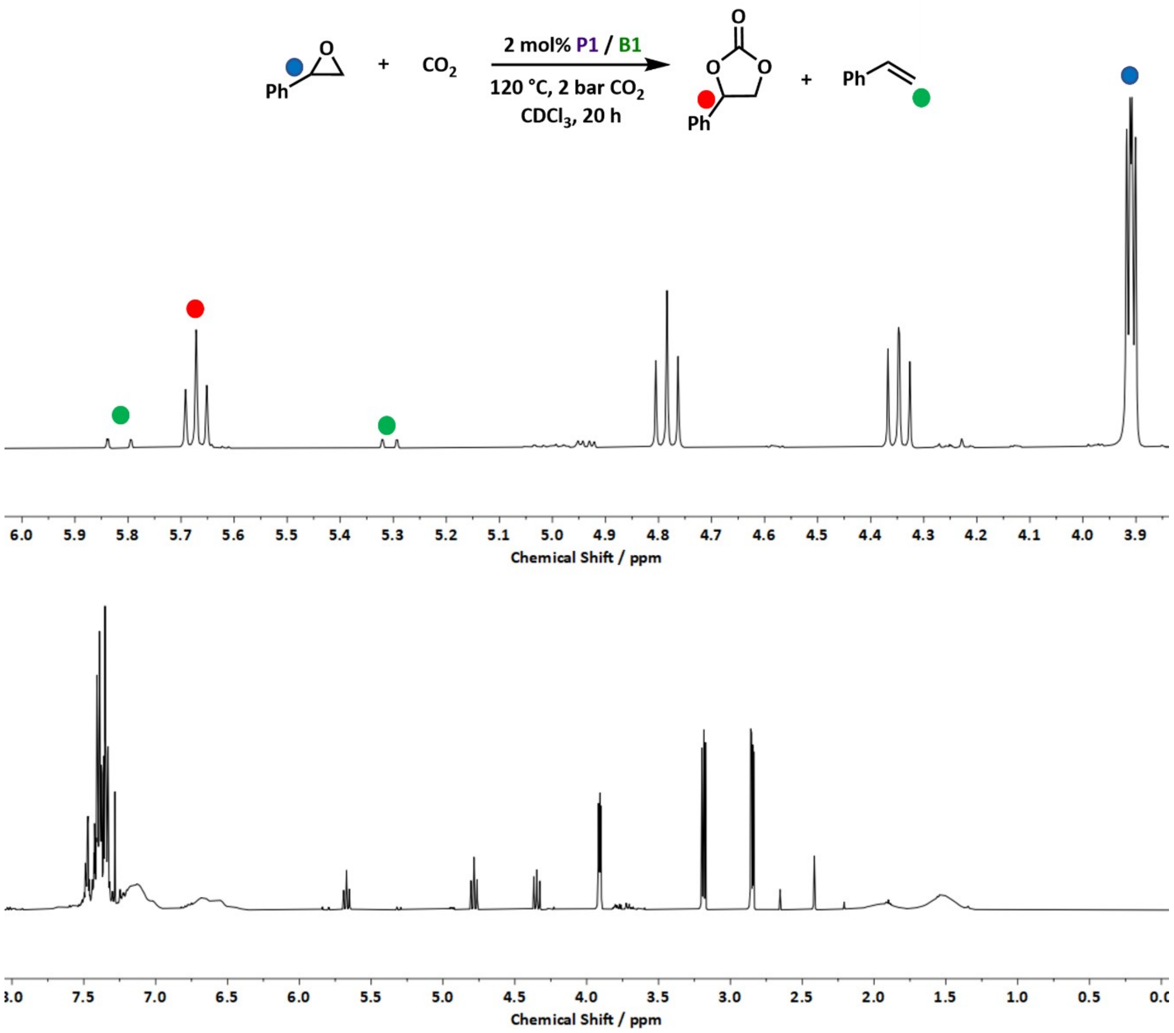

Figure S7: ^1H NMR spectrum of attempted CO_2 insertion to **SO** using a **B1**/**P1** catalytic system (bottom) magnified to show formation of vinyl peaks (top).

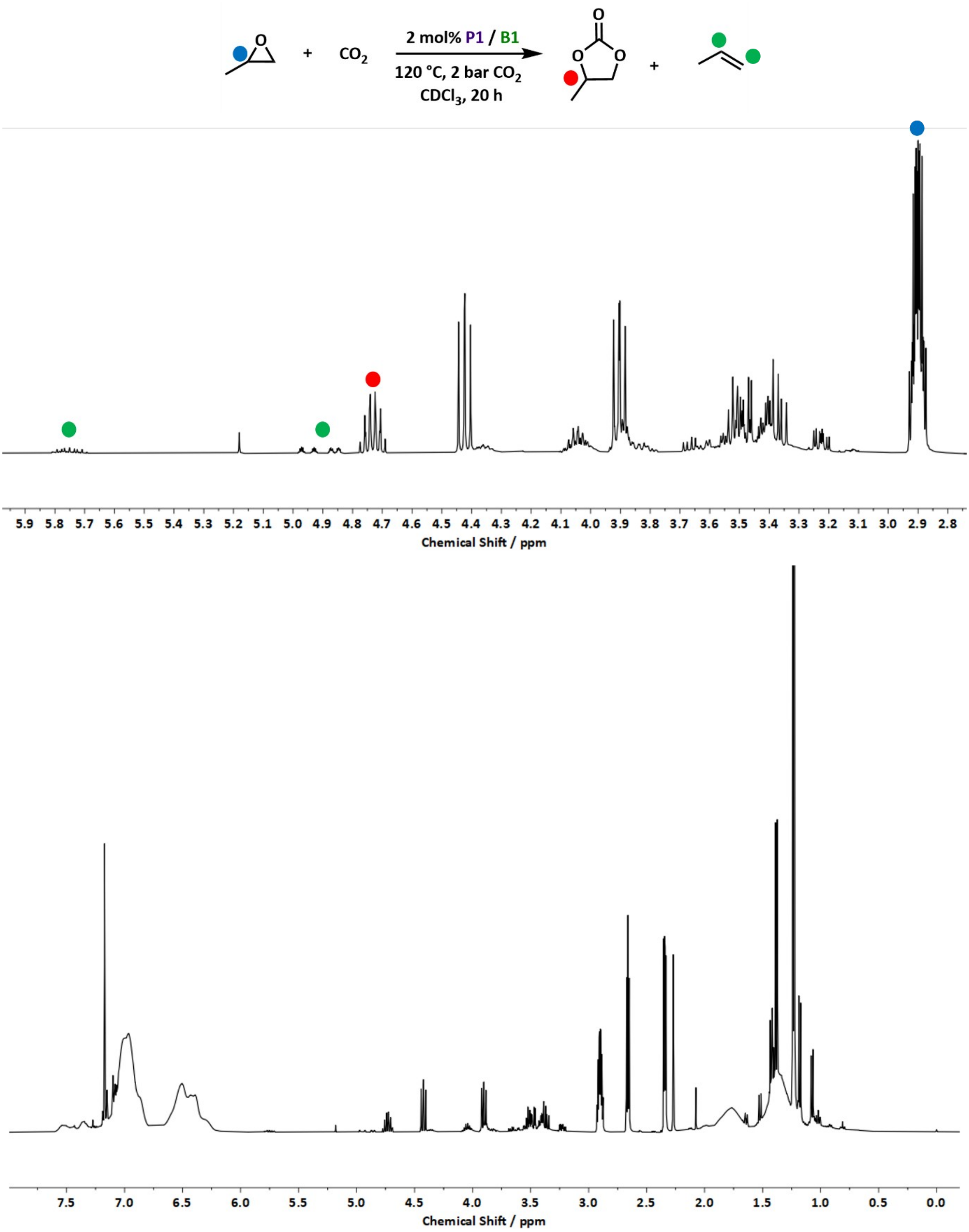

Figure S8: ^{1}H NMR spectrum of attempted CO_2 insertion to **PO** using a **B1**/**P1** catalytic system (bottom), magnified to show formation of vinyl peaks (top). Milder reaction conditions used to monitor reaciton *in situ* led to additional peaks not observed previously believed to correspond to reaction intermediate species.

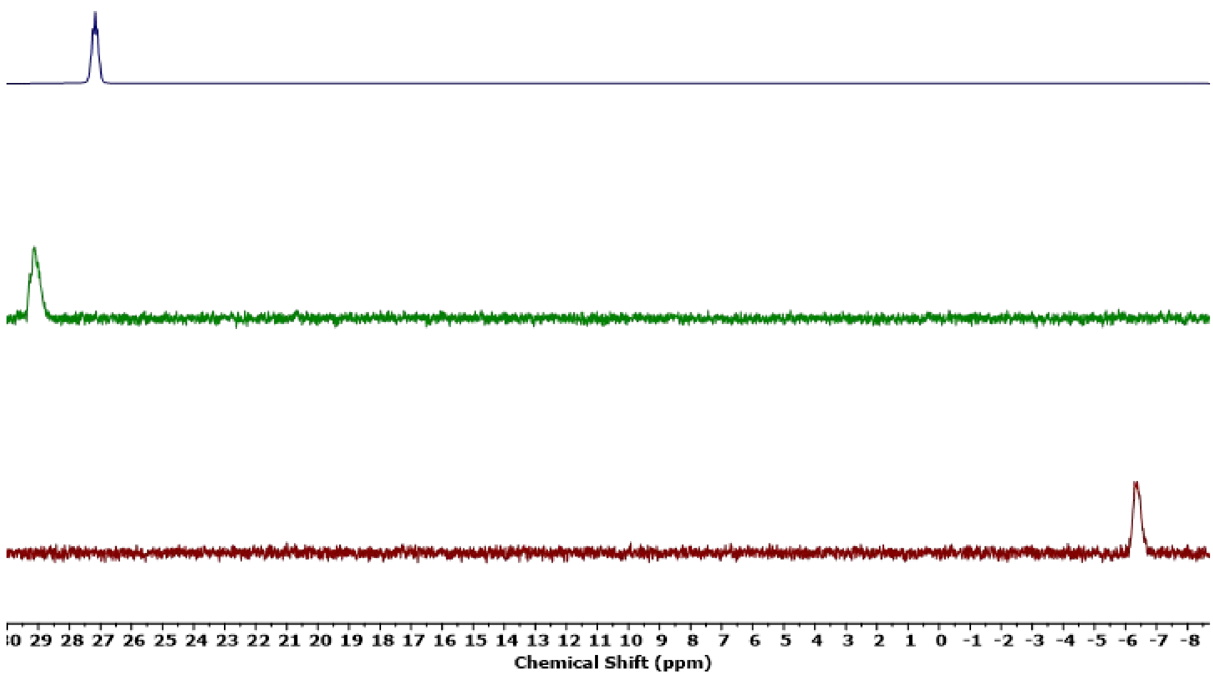

Figure S9: ^{31}P NMR spectrum showing **P1** prior to catalysis with **SO** (bottom), post-reaction (middle) and small molecule Ph_3PO standard for comparison.

Prior to discovering that oxidation can be offset, and selectivity therefore improved, using a **P2**/**B1** system, efforts were made using 1,3-diphenyldisiloxane (**DPDS**) to regenerate the phosphine LB component *in situ*.[6] Indeed, full conversion was obtained when adding 1 equivalent of DPDS to the reaction mixture. In this case, >99% conversion of PO can be obtained, while 2 phosphine species can be observed at the end of the reaction, corresponding to oxidised phosphine and epoxide-activated intermediate:

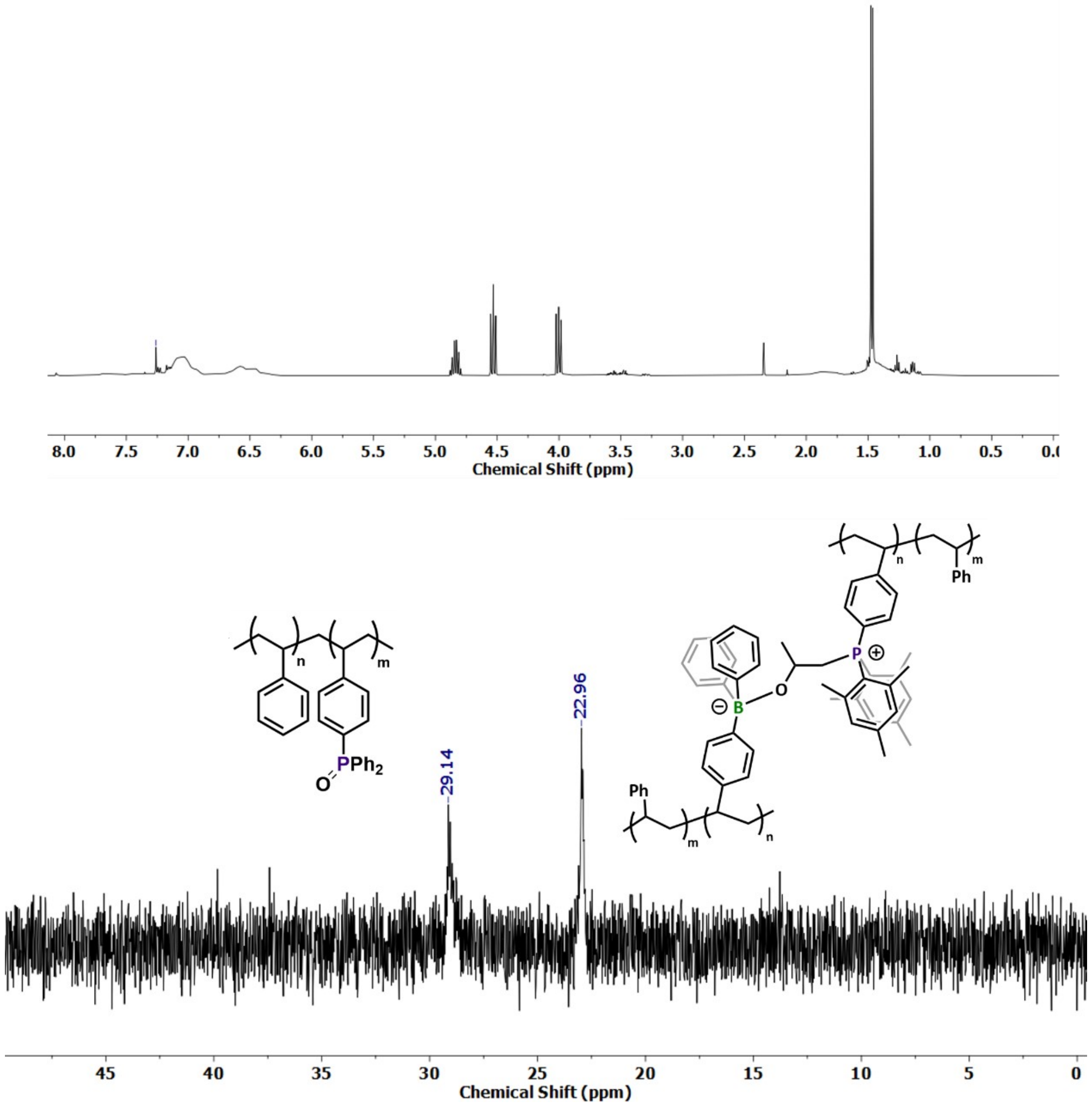

Figure S10: ^{31}P NMR spectrum at >99% conversion for CO_2 insertion to **PO** using a **P1**/**B1** catalytic system in the presence of **DPDS**. Lack of full oxidation and increased conversion shows ability to regenerate *in situ* while revealing catalyst resting state of ring-opened epoxide crosslinked intermediate.

Screening of CO_2 insertion to epoxides using P2/B1

Table S2: Catalytic Data for P2/B1 catalysed reactions of CO2 with PO

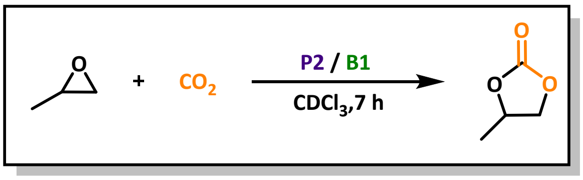

Entry[a]	[epoxide]:[B1]:[P2]	P_{CO_2} (bar)	Temp (°C)	Conversion (%)[b]
1	100:0:0	2	120	-
2	100:1:0	2	120	-
3	100:0:1	2	120	8
4[c]	100:2:2	2	120	-
5[d]	100:2:2	2	120	-
6[e]	100:2:2	2	120	31
7	100:2:2	2	120	>99
8	100:2:2	1	120	81
9	100:2:2	2	100	48
10	100:2:2	2	80	15
11	100:1:1	2	120	19
12	100:0.5:0.5	2	120	4
13	100:1:2	2	120	46
14[f]	100:4:1	2	120	15

[a]General reaction conditions unless otherwise indicated: **PO** (0.8 mmol), **B1/P2** catalytic system, $CDCl_3$ solvent, 1,3,5-trimethoxybenzene internal standard. [b]Determined by ^{1}H NMR spectroscopy. [c]THF used as solvent. [d]Toluene used as solvent. [e] Dichloromethane used as solvent.

Table S3: Catalytic Data for CO_2 insertion to epoxide substrates catalysed by **P2/B1**

Substrate[a]	Conversion % (6 h)[b]	>99% Conversion Within (h)
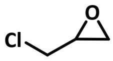	>99	5
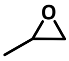	90	7
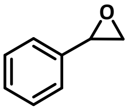	62	20
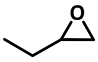	41	24
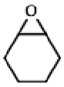	-	-
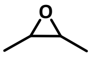	-	-

[a]General reaction conditions unless otherwise indicated: **Epoxide** (0.8 mmol), **B1/P2** catalytic system, $CDCl_3$ solvent, 1,3,5-trimethoxybenzene internal standard. [b]Determined by ^{1}H NMR spectroscopy.

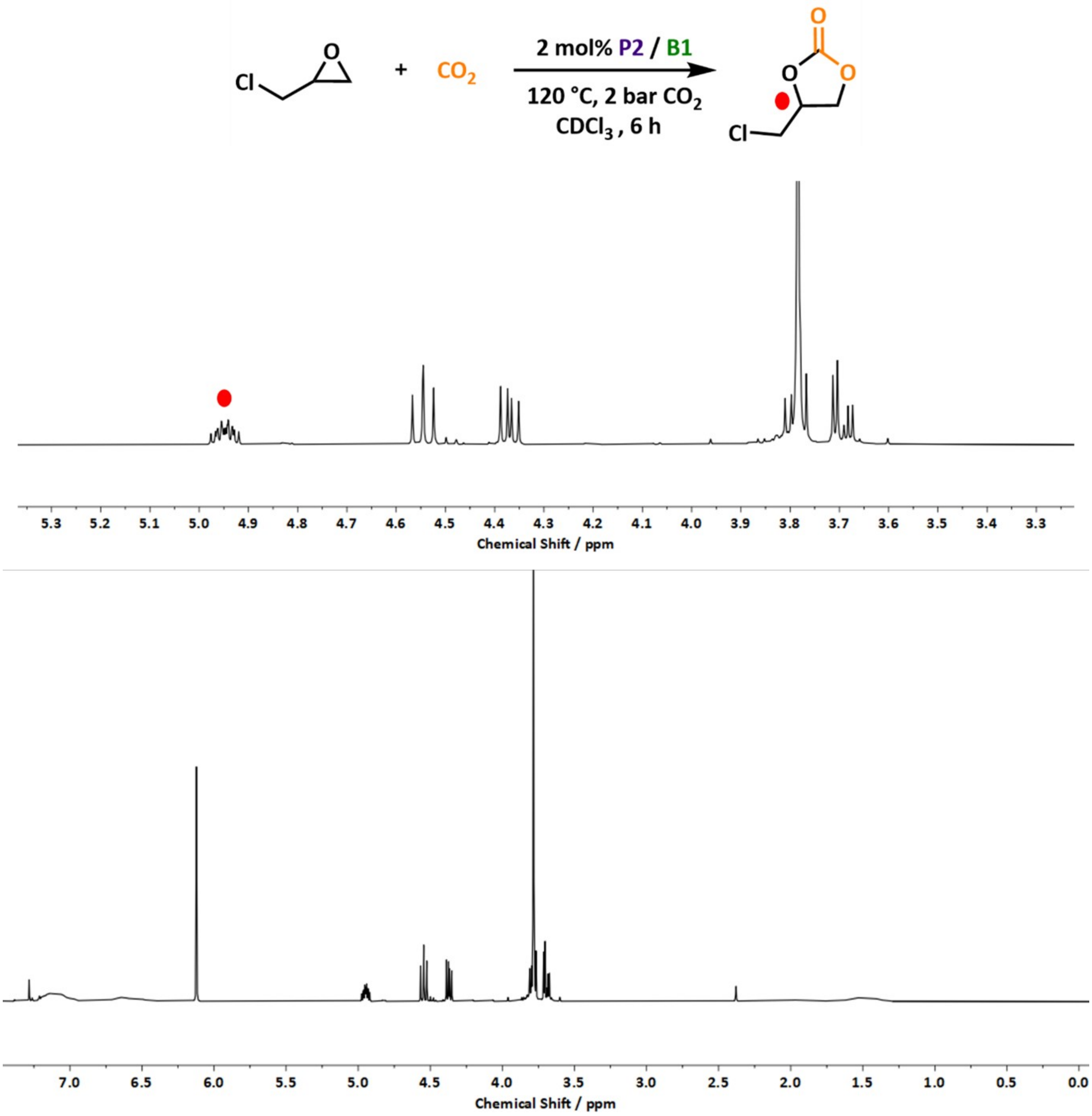

Figure S11: Crude ^{1}H NMR spectra (bottom) and magnified product/reactant region (top) for CO2 insertion to CPO using a **P2**/**B1** catalytic system. Peaks at 6.1 and 3.7ppm correspond to internal standard – 1,3,5-trimethoxybenzene.

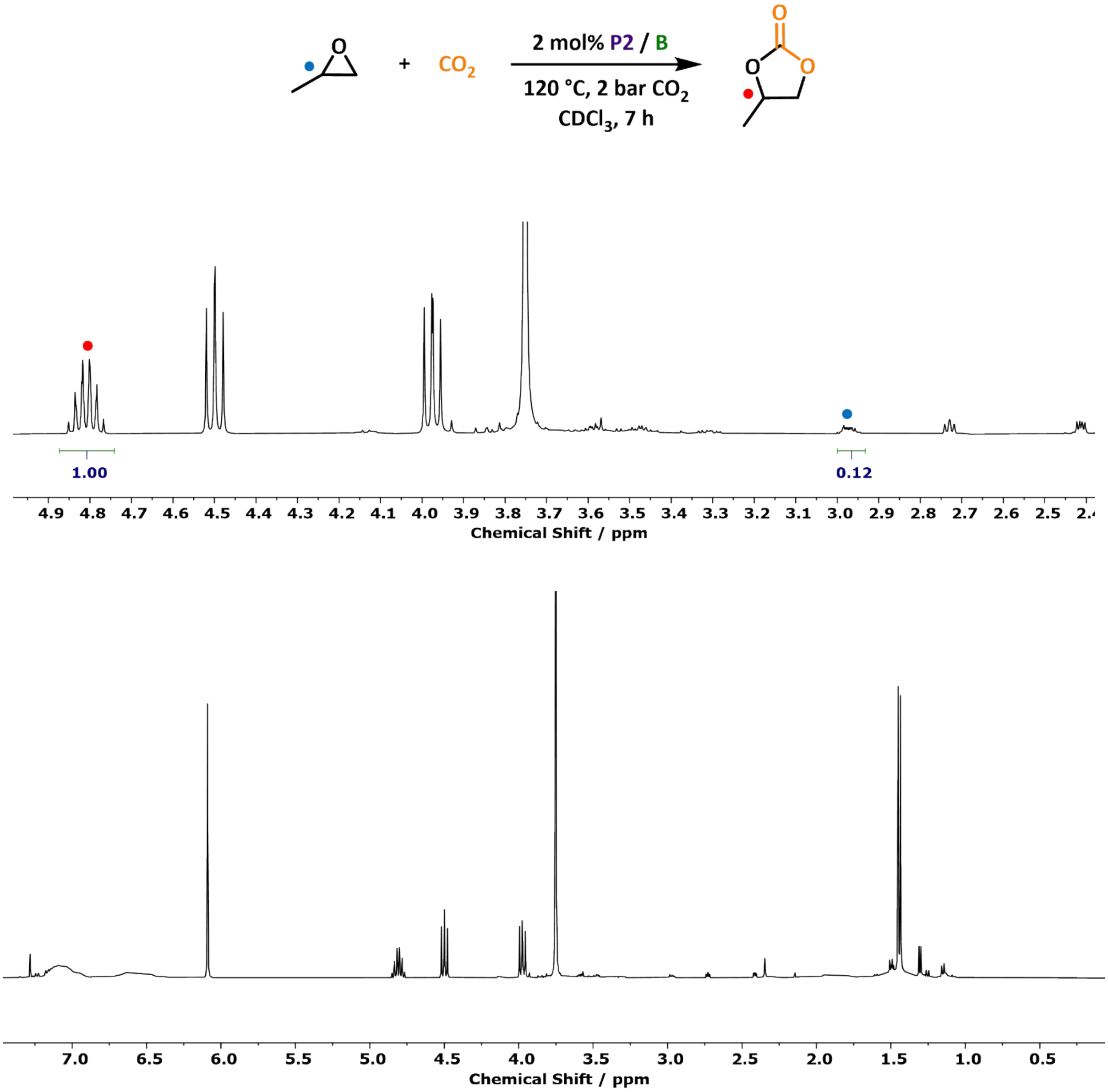

Figure S12: Crude ^{1}H NMR spectra (bottom) and magnified product/reactant region (top) for CO_2 insertion to **PO** using a **P2**/**B1** catalytic system. Peaks at 6.1 and 3.7ppm correspond to internal standard – 1,3,5-trimethoxybenzene.

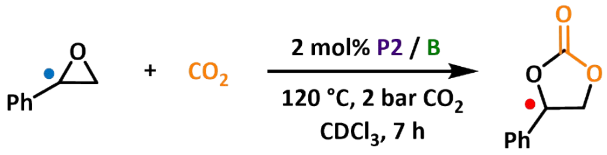

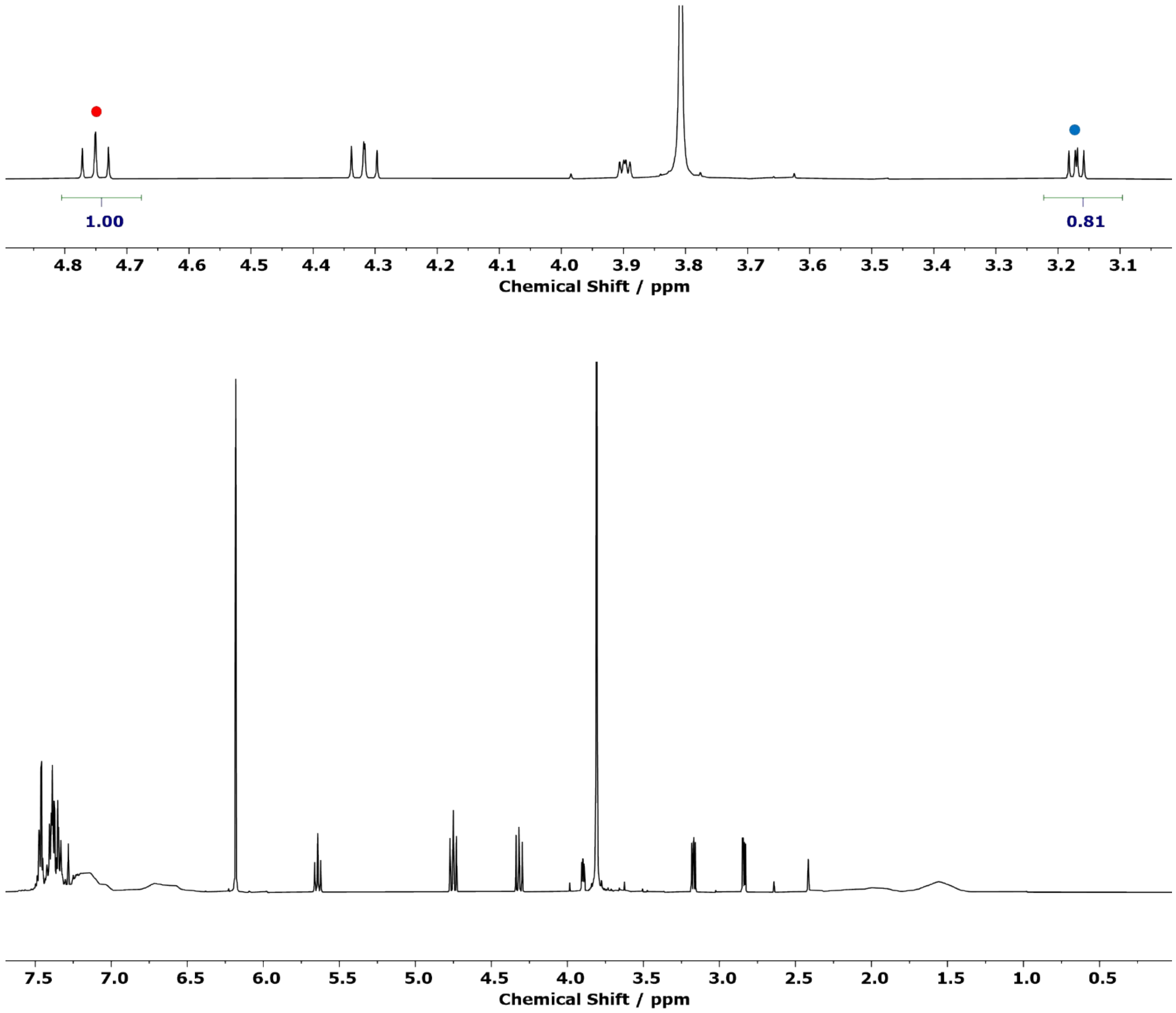

Figure S13: Crude ^{1}H NMR spectra (bottom) and magnified product/reactant region (top) for CO_2 insertion to **SO** using a **P2/B1** catalytic system. Peaks at 6.1 and 3.7ppm correspond to internal standard – 1,3,5-trimethoxybenzene.

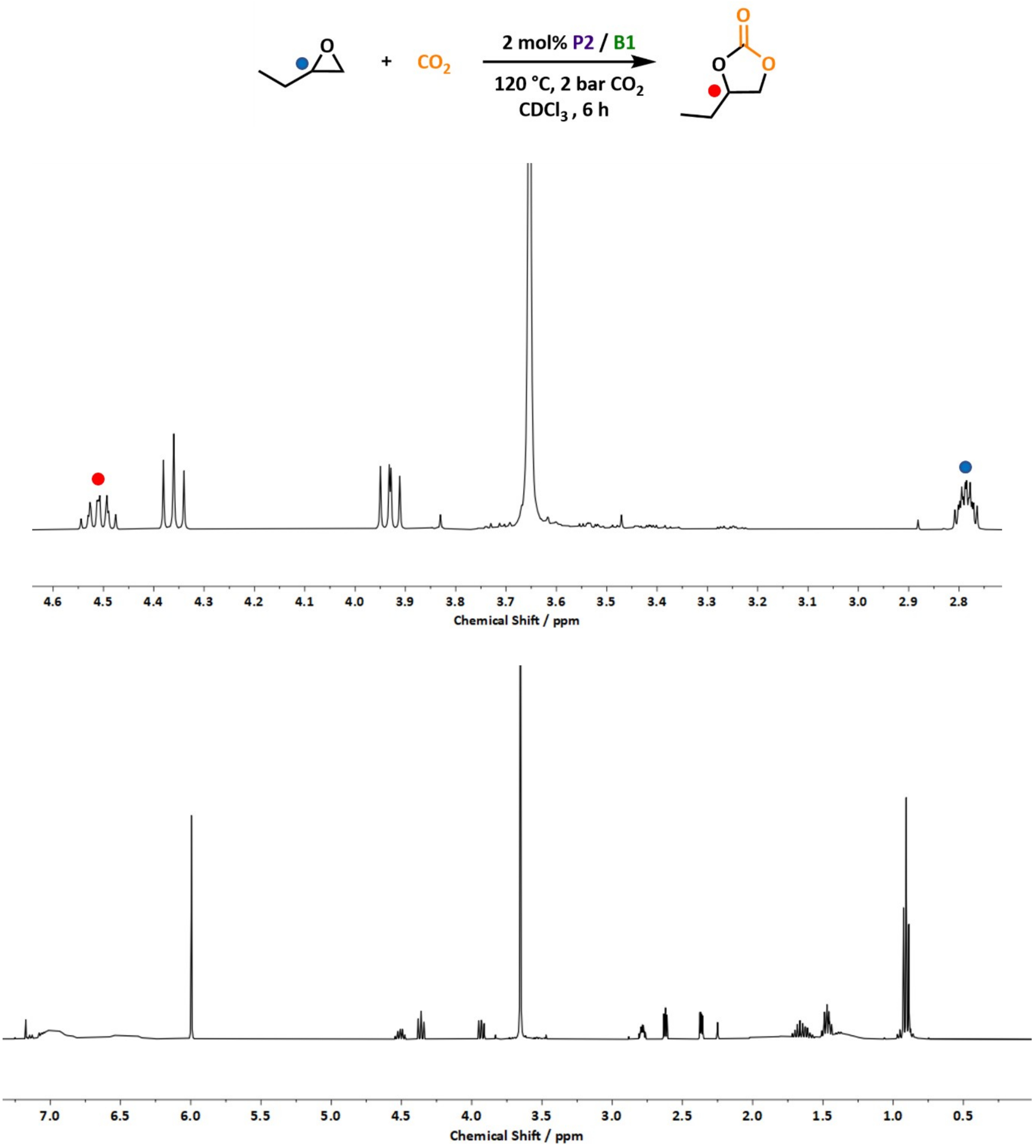

Figure S14: Crude ^{1}H NMR spectra (bottom) and magnified product/reactant region (top) for CO_2 insertion to **BO** using a **P2**/**B1** catalytic system. Peaks at 6.1 and 3.7ppm correspond to internal standard – 1,3,5-trimethoxybenzene.

4. Mechanistic Investigations

In order to better understand the route of oxidation present when using **P1**, small molecule model compounds were synthesised using **BPh_3** and **PPh_3** as the LA and LB components respectively. Since oxidation was established to be an on-cycle process, we focussed on peaks corresponding to the opened cyclic ether in both cases. In order to assess the impact of switching to **P2**, a small molecule model compound using **BPh_3** and **$PMes_2Ph$** was also synthesised.

P1 small molecule model systems

Activation of propylene oxide, PO:

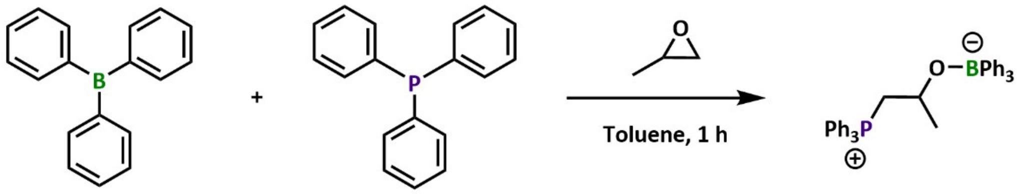

In a glovebox, solutions of triphenyl borane (0.150 g, 0.620 mmol) and triphenylphosphine (0.162 g, 0.619 mmol) in toluene were combined to give a total volume of 3 mL and stirred at room temperature. Then, propylene oxide (0.036 g,0.619 mmol) in toluene (1 mL) was added across 5 minutes and the suspension stirred for an hour. Afterwards, the formed solid was collected by filtration and rinsed with n-hexane to obtain the desired product as a white powder in 83% yield (0.29 g).

Peaks corresponding to ring-opened epoxide are as follows:

^{1}H NMR (400 MHz, 294 K, DCM-d$_2$) δ / ppm 4.05 – 3.93 (m, 1H, OCH(CH$_3$)), 3.11 (ddd, $J_{P,H}$ = 15.3 Hz, $J_{H,H}$ = 12.9, 6.0 Hz, 1H, P-CH*H*), 2.74 (ddd, $J_{P,H}$ = 15.3 Hz, $J_{H,H}$ = 11.4, 5.6 Hz, 1H, P-C*H*H), 1.11 (dd, *J* = 6, 1.5 Hz, 3H, CH$_3$)

^{13}C{^{1}H} NMR (126 MHz, 294 K, DCM-d$_2$) δ / ppm 64.44 (d, $J_{P,C}$ = 3.9 Hz, O-CH), 33.90 (d, $J_{P,C}$ = 43.0 Hz, P-CH$_2$), 26.01 (d, $J_{P,C}$ = 7.8 Hz, CH$_3$)

^{11}B NMR (128 MHz, 295K, DCM-d$_2$) δ / ppm 1.34 (br)

^{31}P{^{1}H} NMR (162 MHz, 295 K, DCM-d$_2$) δ / ppm 22.36 (s)

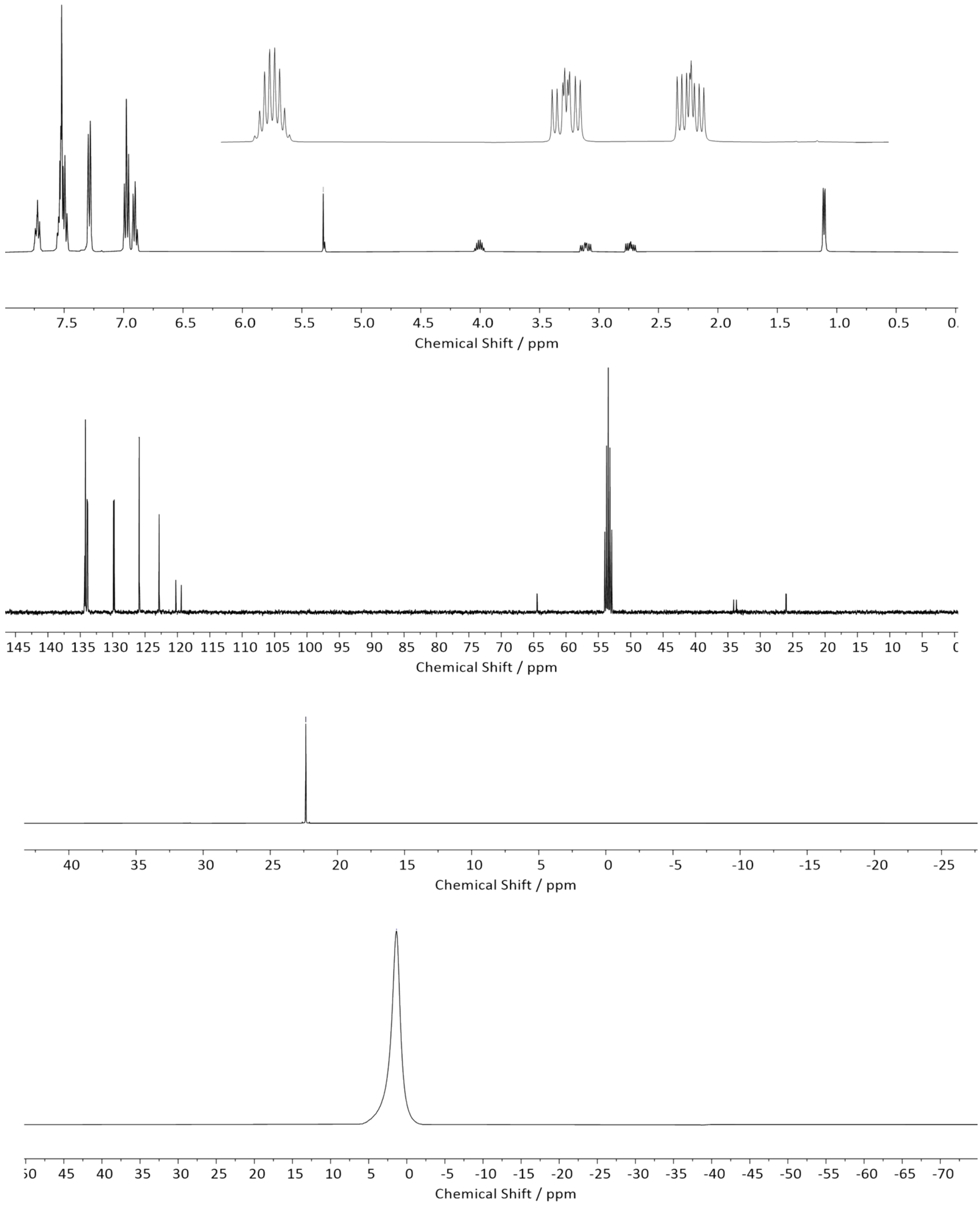

Figure S15: ^{1}H, ^{13}C, ^{31}P and ^{11}B (top to bottom) NMR spectroscopic analysis of epoxide activated intermediate present in catalytic cycle when **PO** is used as a substrate with a **P1**/**B1** catalytic system.

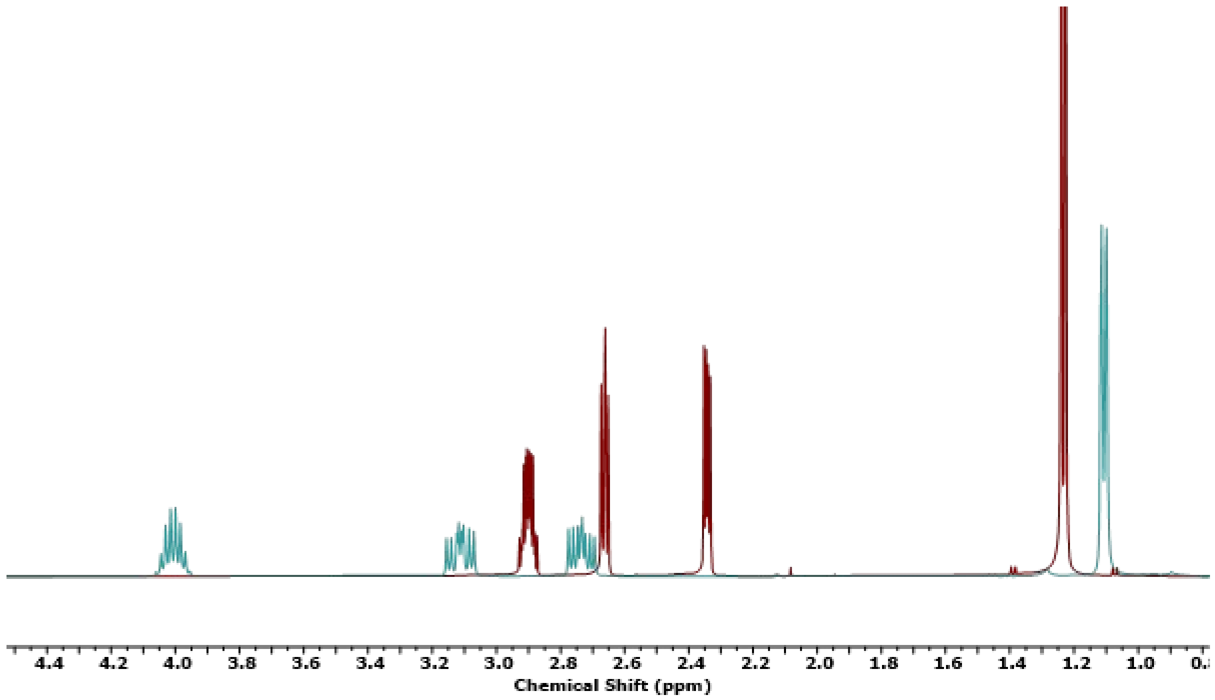

Figure S16: Superimposed spectra showing impact of epoxide activation by **P1/B1**. Red = Propylene oxide, Blue = SM complex of ring-opened epoxide.

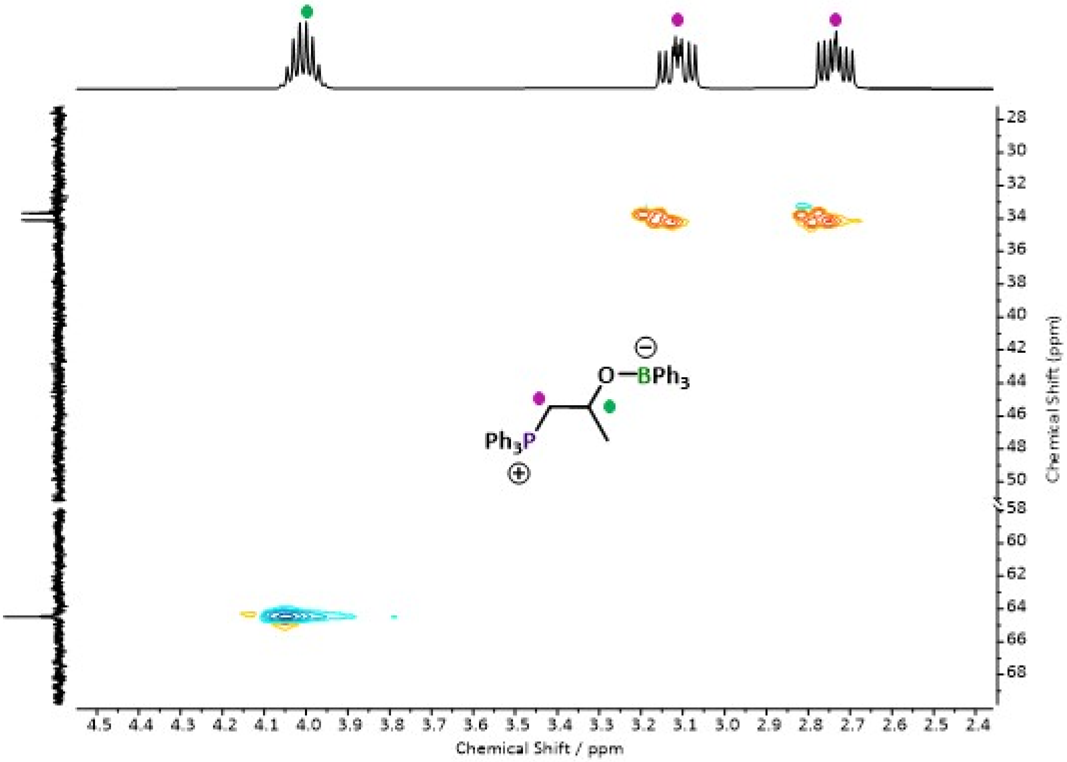

Figure S17: HSQC analysis demonstrating position of LB attack when **PO** is used as a substrate with a **P1**/**B1** catalyst system. Red = CH_2, Blue = CH or CH_3. Using ^{31}P-^{13}C coupling constants, attack of **P1** therefore occurs at the least hindered carbon. Solvent peaks omitted for clarity.

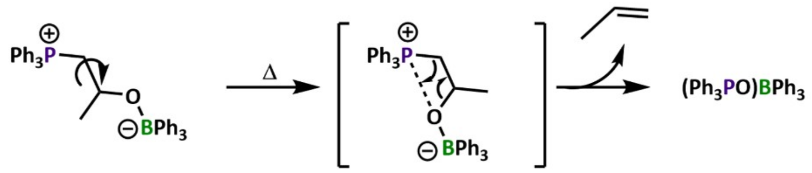

I II

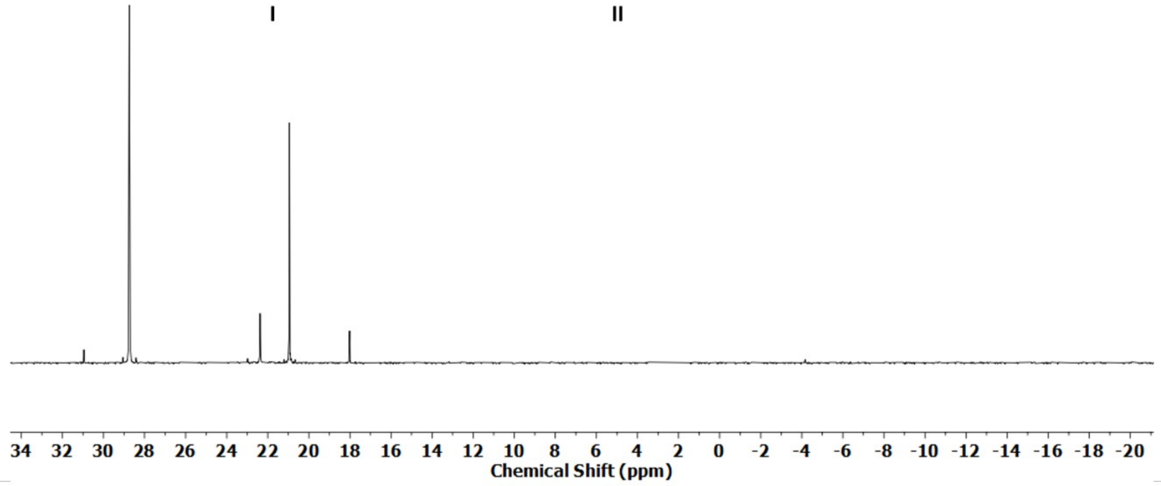

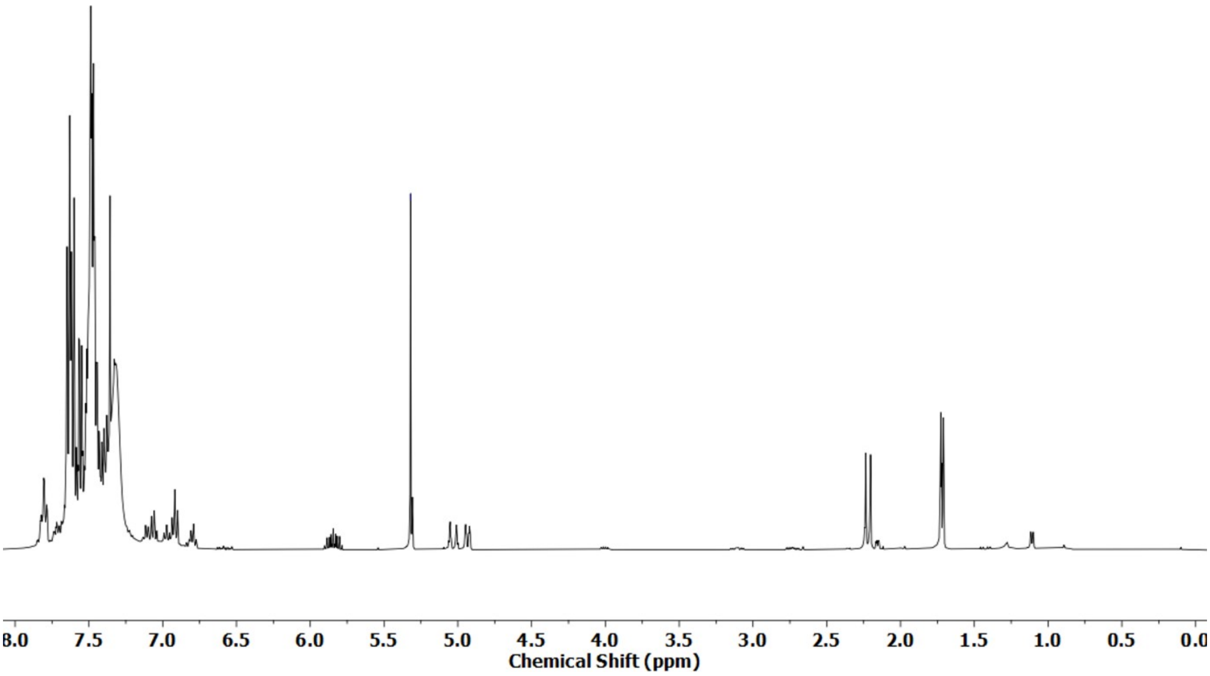

Figure S18: ^{31}P (top) and ^{1}H (bottom) NMR spectra showing heated activated **PO** complex, **PPh_3.PO.BPh_3** (**I**). Clear formation of propene can be observed, in addition to phosphine oxidation via species 2.

Activation of styrene oxide, SO

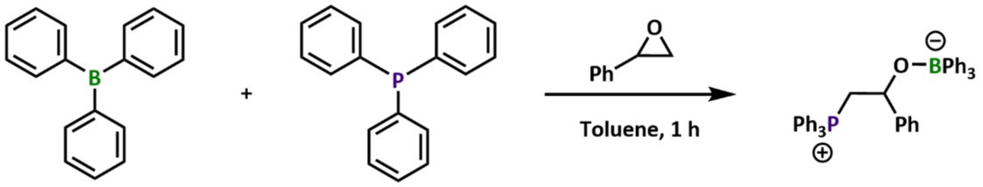

Triphenylphosphine (0.162 g, 0.619 mmol) and triphenylborane (0.150 g, 0.620 mmol) were mixed in toluene (3 mL) at r.t. Then, styrene oxide (**SO**, 0.0744 g, 0.619 mmol, as a stock solution in 1 mL of toluene) was added dropwise in 10 min and the mixture was stirred for another 50 min. Then, the mixture was precipitated into hexane and the product was collected by filtration. Yield: 0.284 g, 73.5 %.

Peaks corresponding to ring-opened epoxide are as follows:

^{1}H NMR (400 MHz, 294 K, DCM-d$_2$) δ / ppm 4.54-4.44 (m, 1H, P-C*H*), 4.04 (ddd, $J_{P,H}$ = 35.3 Hz, $J_{H,H}$ = 11.5, 5.4 Hz, 1H, O-CH*H*) 3.86 (ddd, $J_{P,H}$ = 13.8 Hz, $J_{H,H}$ = 11.6 Hz, 9.6 Hz, 1H, O-C*H*H)

^{13}C{^{1}H} NMR (126 MHz, 294 K, DCM-d$_2$) δ / ppm 65.08 (d, $J_{P,C}$ = 4.9 Hz, O-CH$_2$), 48.28 (d, $J_{P,C}$ = 44.5 Hz, P-CH)

^{11}B NMR (128 MHz, 295K, DCM-d$_2$) δ / ppm 2.07 (br)

^{31}P{^{1}H} NMR (162 MHz, 295 K, DCM-d$_2$) δ / ppm 25.37 (s)

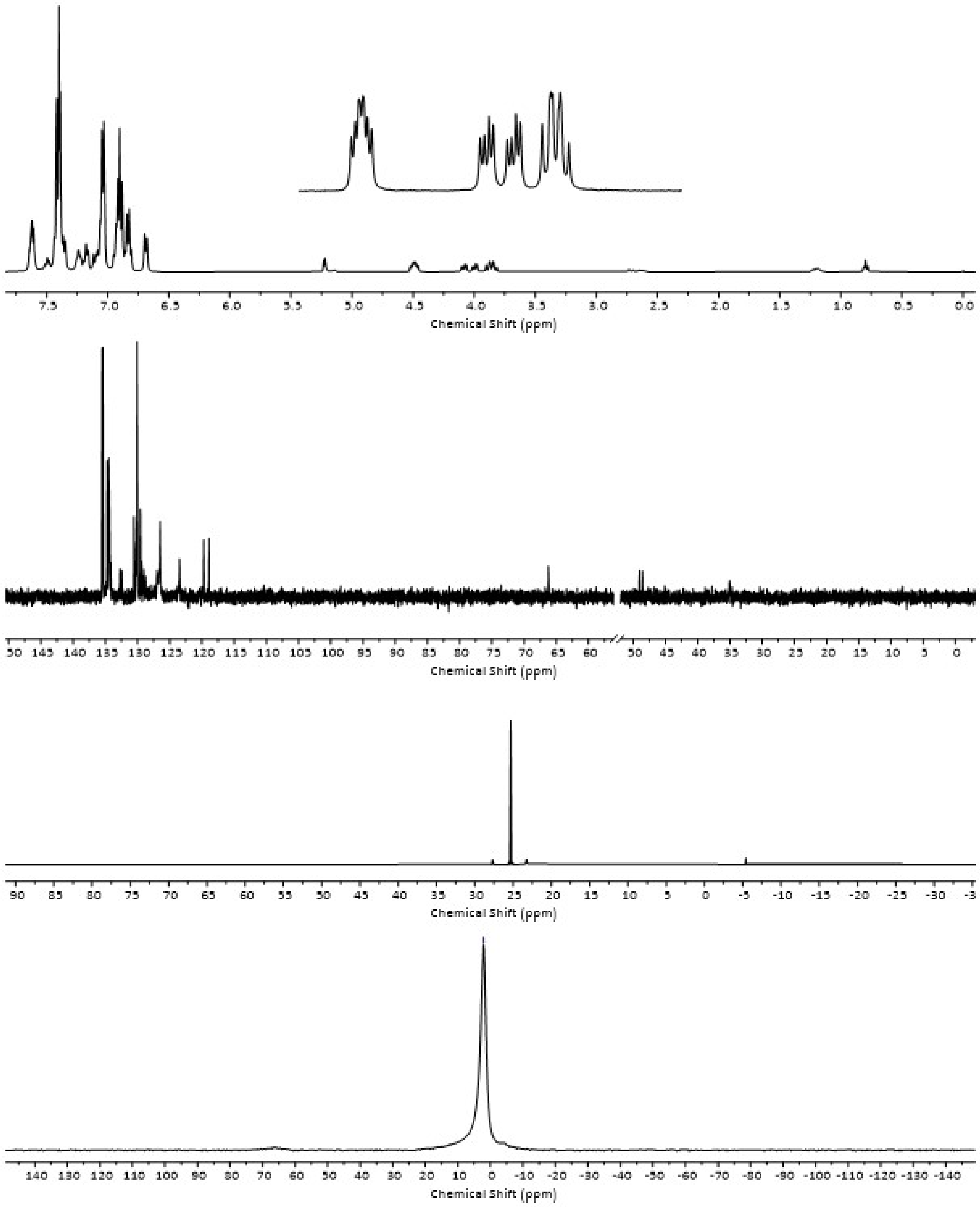

Figure S19: ^{1}H, ^{13}C (solvent peaks omitted for clarity), ^{31}P and ^{11}B (top to bottom) NMR spectroscopic analysis of epoxide activated intermediate present in catalytic cycle when **PO** is used as a substrate with a **P1**/**B1** catalyst system.

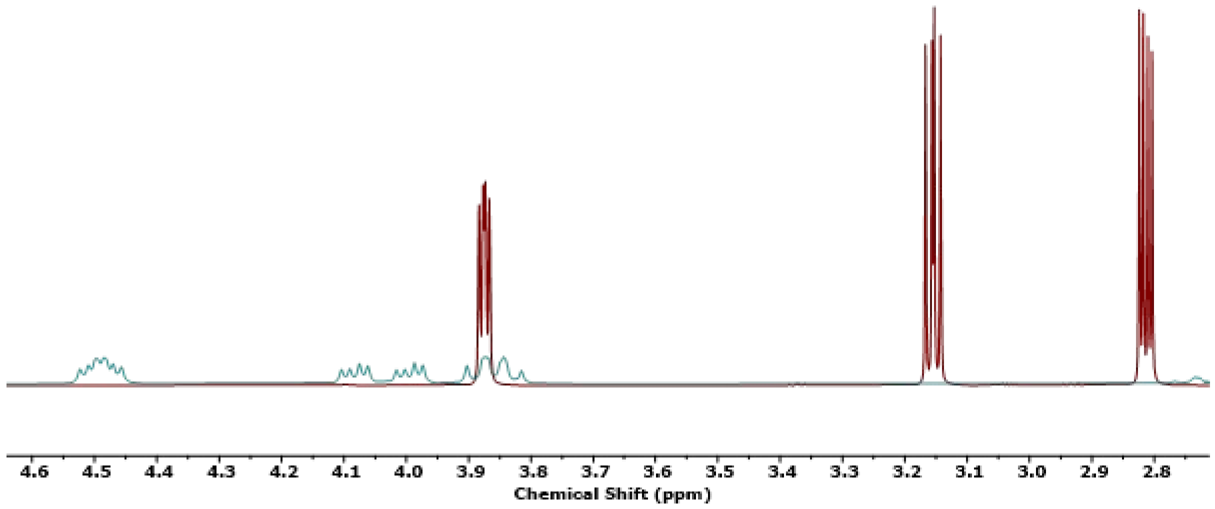

Figure S20: Superimposed spectra showing impact of epoxide activation by **P1/B1**. Red = Styrene oxide (**SO**), Blue = SM complex of ring-opened epoxide.

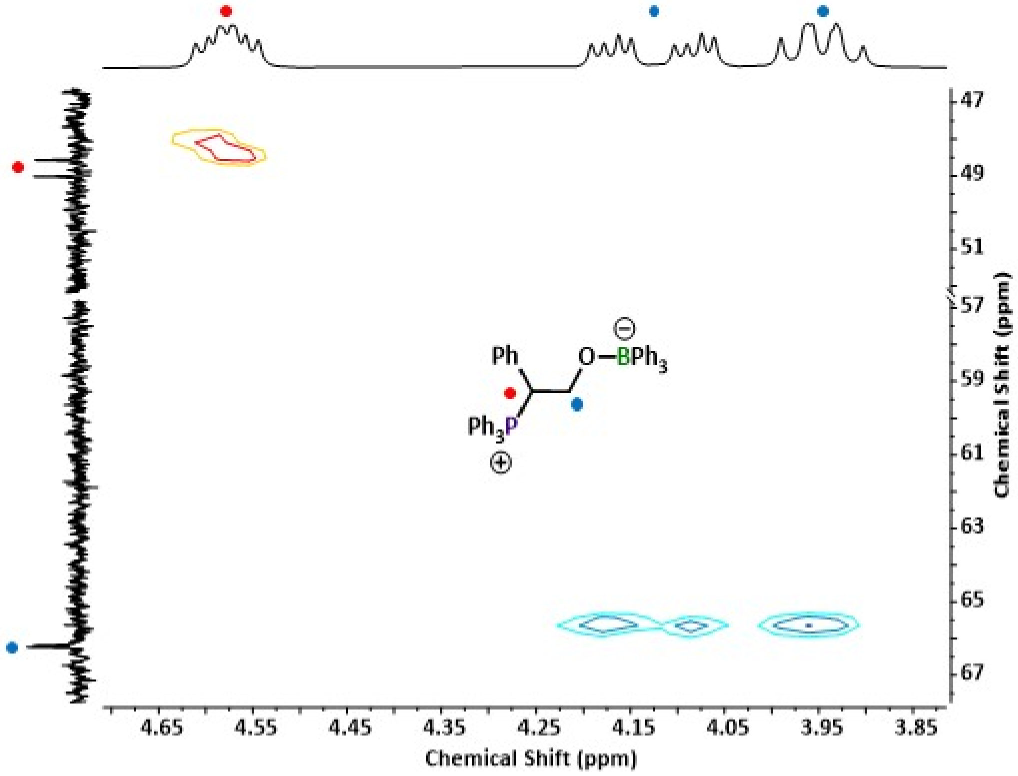

Figure S21: HSQC analysis demonstrating position of LB attack when **SO** is used as a substrate with a **P1/B1** catalyst system. Red = CH_2, Blue = CH or CH_3. Using ^{31}P-^{13}C coupling constants, attack of **P1** therefore occurs at the more hindered carbon.

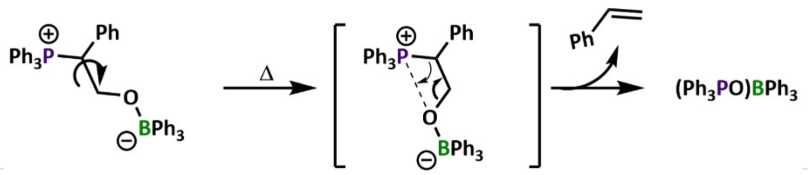

I II

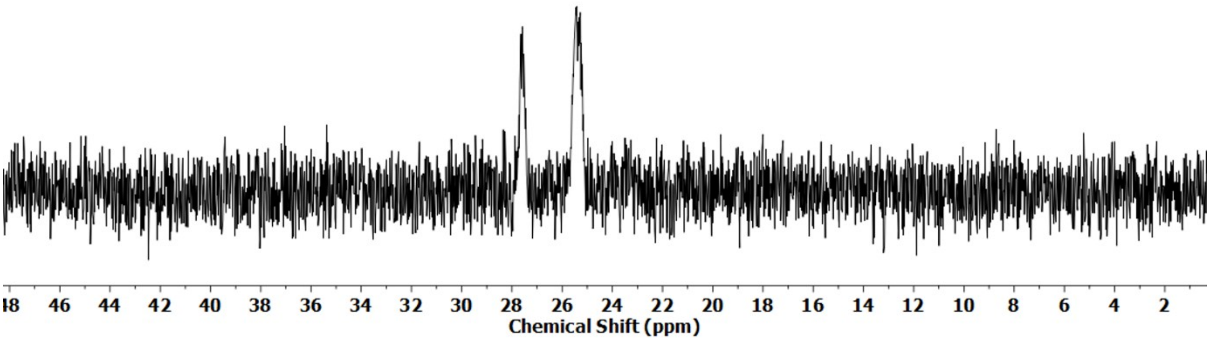

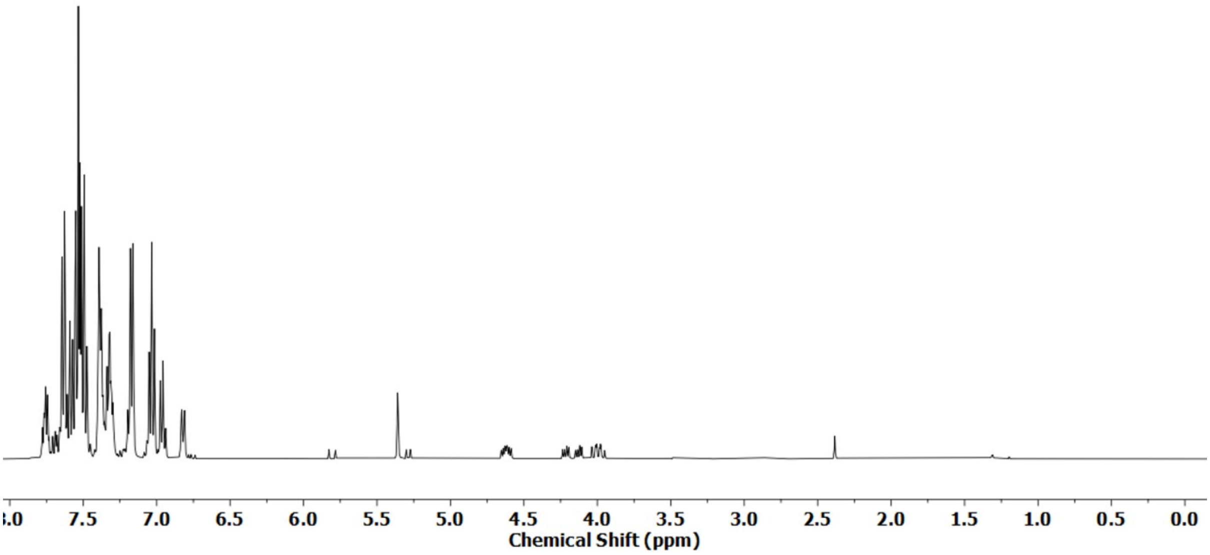

Figure S22: ^{31}P (top) and ^{1}H (bottom) NMR spectra showing heated activated **SO** complex, **PPh_3.SO.BPh_3**. Clear formation of styrene can be observed, in addition to phosphine oxidation.

P2 Small molecule model systems

Activation of propylene oxide, PO

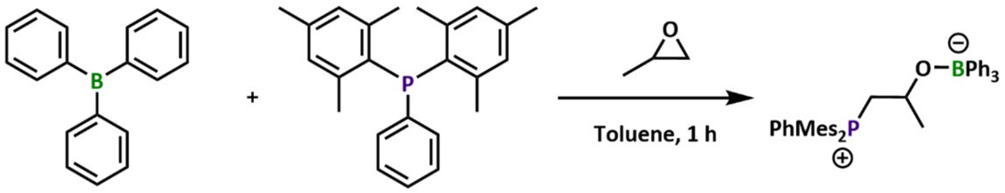

In a glovebox, solutions of triphenyl borane (0.150 g, 0.620 mmol) and dimesitylphenyl phosphine (0.215 g, 0.619 mmol) in toluene were combined to give a total volume of 3 mL and stirred at room temperature. Then, propylene oxide (0.036 g,0.619 mmol) in toluene (1 mL) was added across 5 minutes and the suspension stirred for an hour. Afterwards, the formed solid was collected by filtration and rinsed with n-hexane to obtain the desired product as a white powder in 74% yield (0.30 g).

Peaks corresponding to ring-opened epoxide are as follows:

^{1}H NMR (400 MHz, 294 K, DCM-d_2) δ / ppm 3.68 – 3.55 (m, 2H, O-C*H*H, P-C*H*), 3.20 – 3.06 (m, 1H, O-CH*H*), 0.93 (dd, J_{HH} = 5.8 Hz, 2.8 Hz, 3H)

^{13}C{^{1}H} NMR (126 MHz, 294 K, DCM-d_2) δ / ppm 65.91 (d, $J_{P,C}$ = 3.4 Hz, O-CH), 41.98 (d, $J_{P,C}$ = 45.0 Hz, P-CH_2), 25.67 (d, $J_{P,C}$ = 14.7 Hz)

^{11}B NMR (128 MHz, 295K, DCM-d_2) δ / ppm 1.31 (br)

^{31}P{^{1}H} NMR (162 MHz, 295 K, DCM-d_2) δ / ppm 19.86 (s)

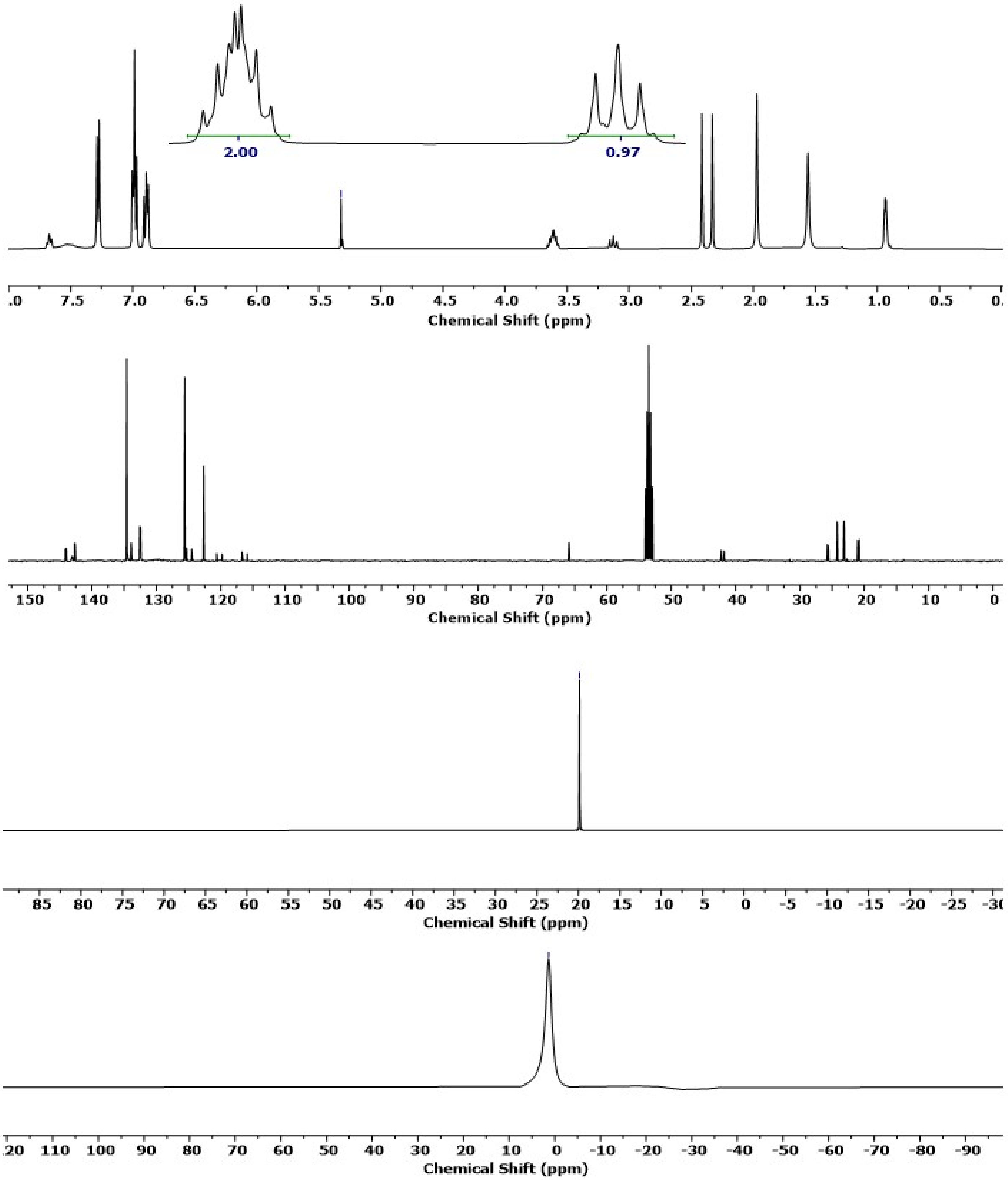

Figure S23: ^{1}H, ^{13}C, ^{31}P and ^{11}B (top to bottom) NMR spectroscopic analysis of epoxide activated intermediate present in catalytic cycle when **PO** is used as a substrate with a **P2**/**B1** catalyst system.

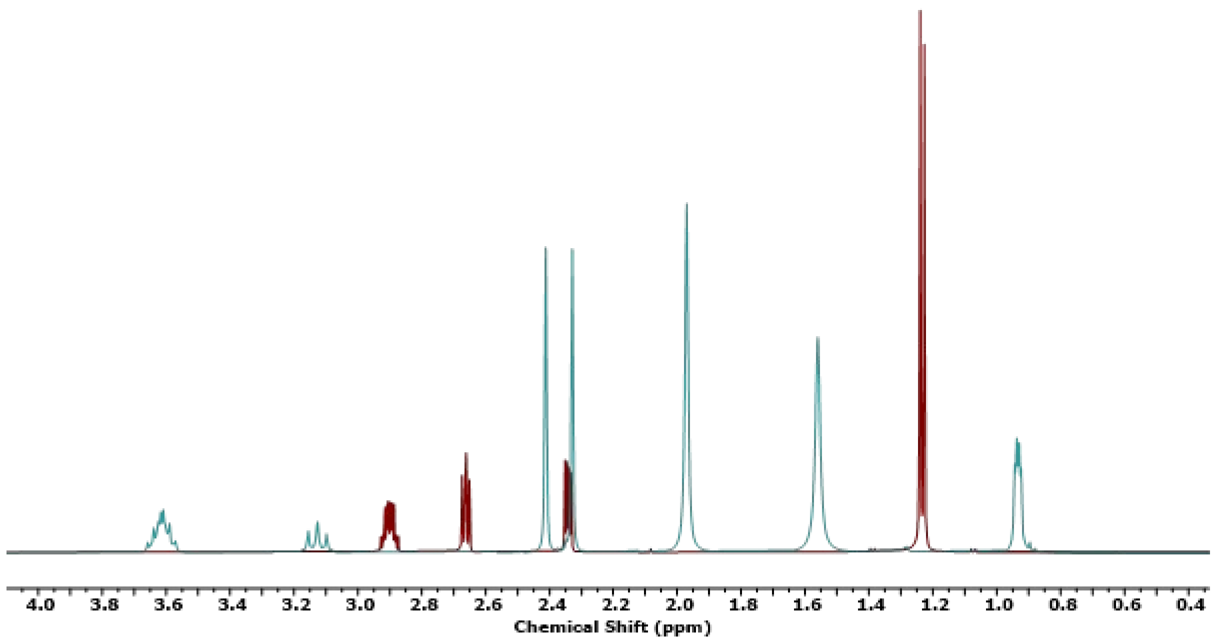

Figure S24: Superimposed spectra showing impact of epoxide activation by **P2/B1**. Red = Propylene oxide oxide (**PO**), Blue = SM complex of ring-opened epoxide.

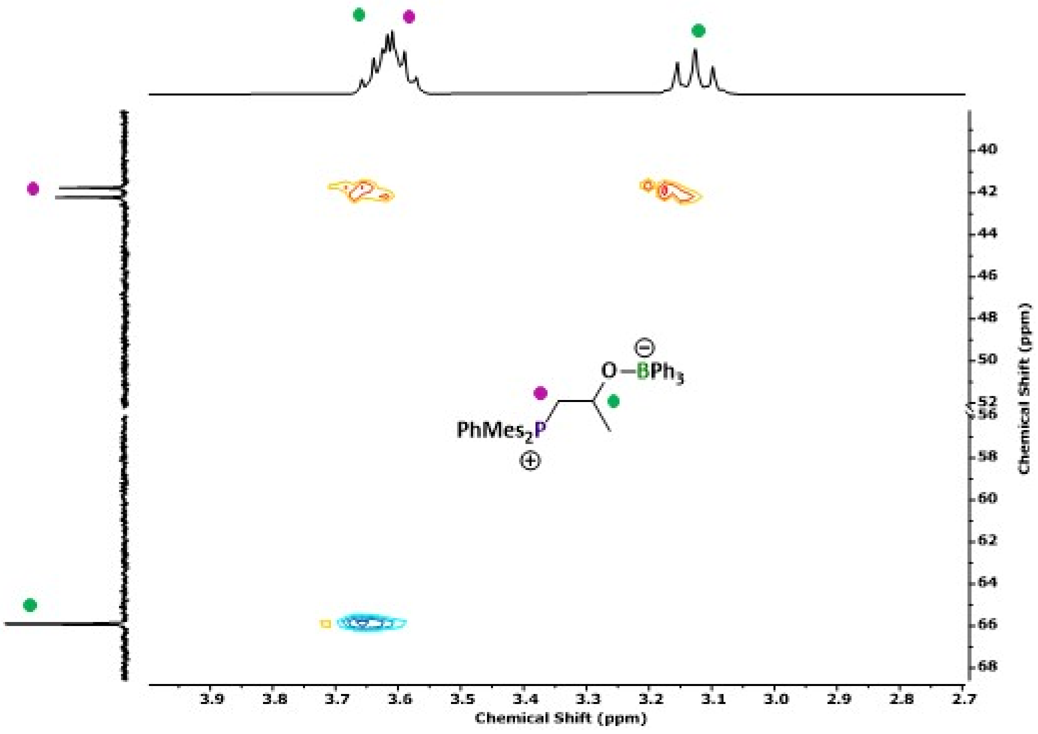

Figure S25: HSQC analysis demonstrating position of LB attack when **PO** is used as a substrate with a **P2/B1** catalyst system. Red = CH2, Blue = CH or CH_3. Overlap of O-$CHCH_3$ and P-CHH protons can be observed. Using ^{1}P-^{13}C coupling constants, attack of P1 therefore occurs at the more hindered carbon. Solvent peaks omitted for clarity.

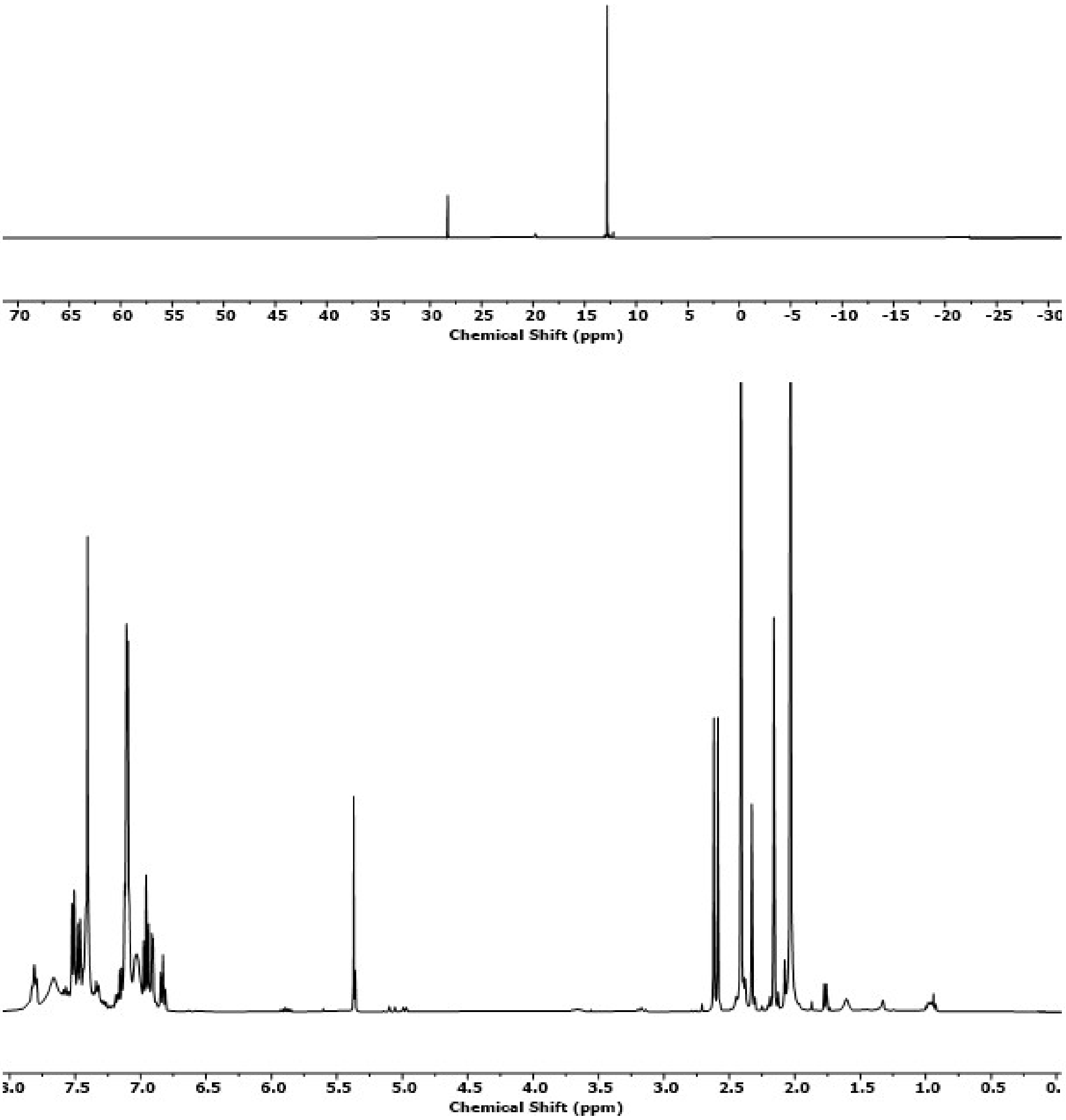

Figure S26: ^{31}P (top) and ^{1}H (bottom) NMR spectra showing heated activated **PO** complex, **PMes$_2$Ph.PO**.**BPh$_3$**. Some propene and oxidised phosphine formation can be observed, while other forms of decomposition dominate.

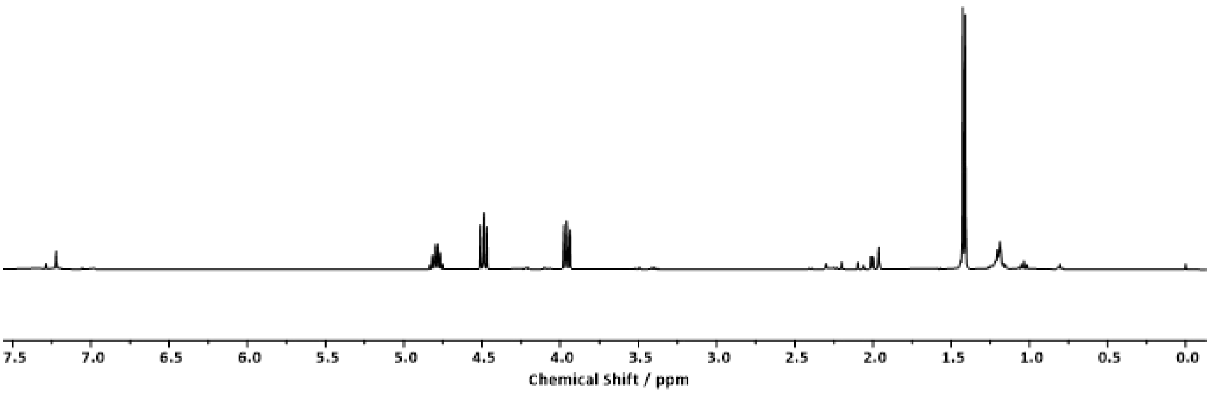

Figure S27: Crude ^{1}H NMR spectra for CO_2 insertion to **PO** using a $PMes_2Ph$ BPh_3 catalytic system with 6 hour reaction time. Despite alkene product release from heating of small molecule model compound, no vinyl product formation is observed and quantitative conversion is obtained.

Activation of styrene oxide, SO

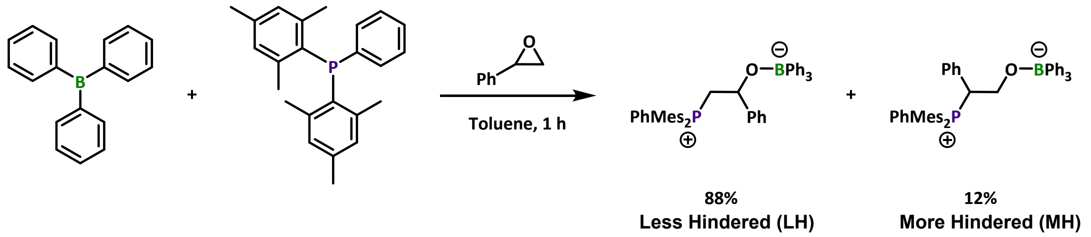

Dimesitylphenylphosphine (0.215 g, 0.619 mmol) and triphenylborane (0.150 g, 0.620 mmol) were mixed in toluene (3 mL) at r.t. Then, styrene oxide (**SO**, 0.0744 g, 0.619 mmol, as a stock solution in 1 mL of toluene) was added dropwise in 10 min and the mixture was stirred for another 50 min. Then the mixture was precipitated into hexane and the product collected by filtration. Yield: 0.297 g, 67.6%.

Peaks corresponding to ring-opened epoxide are as follows:

LH:

^{1}H NMR (400 MHz, 294 K, DCM-d$_2$) δ / ppm 4.65 (ddd, J_{PH} = 10.8 Hz, J_{HH} = 7.9, 3.6 Hz, 1H, O-CH), 3.94 – 3.82 (m, 1H, P-C*H*H), 3.53 – 3.40 (m, 1H, P-CH*H*)

^{13}C{^{1}H} NMR (126 MHz, 294 K, DCM-d$_2$) δ / ppm 73.9 (s, O-CH), 41.64 (d, $J_{P,C}$ = 38.6 Hz)

^{11}B NMR (128 MHz, 295K, DCM-d$_2$) δ / ppm 2.09 (br)

^{31}P{^{1}H} NMR (162 MHz, 295 K, DCM-d$_2$) δ / ppm 15.05 (s, **LH**)

MH:

^{1}H NMR (400 MHz, 294 K, DCM-d$_2$) δ / ppm 5.10 (ddd, J_{PH} = 14.1 Hz, J_{HH} = 8.0, 3.0 Hz, 1H, P-C*H*), 3.73 – 3.56 (m, 2H, O-CH$_2$)

^{13}C{^{1}H} NMR (126 MHz, 294 K, DCM-d$_2$) δ / ppm 69.06 (d, $J_{P,C}$ = 4.9 Hz, O-CH$_2$), 52.02 (d, $J_{P,C}$ = 36.7 Hz, P-CH)

^{11}B NMR (128 MHz, 295K, DCM-d$_2$) δ / ppm 2.09 (br)

^{31}P{^{1}H} NMR (162 MHz, 295 K, DCM-d$_2$) δ / ppm 18.25 (s)

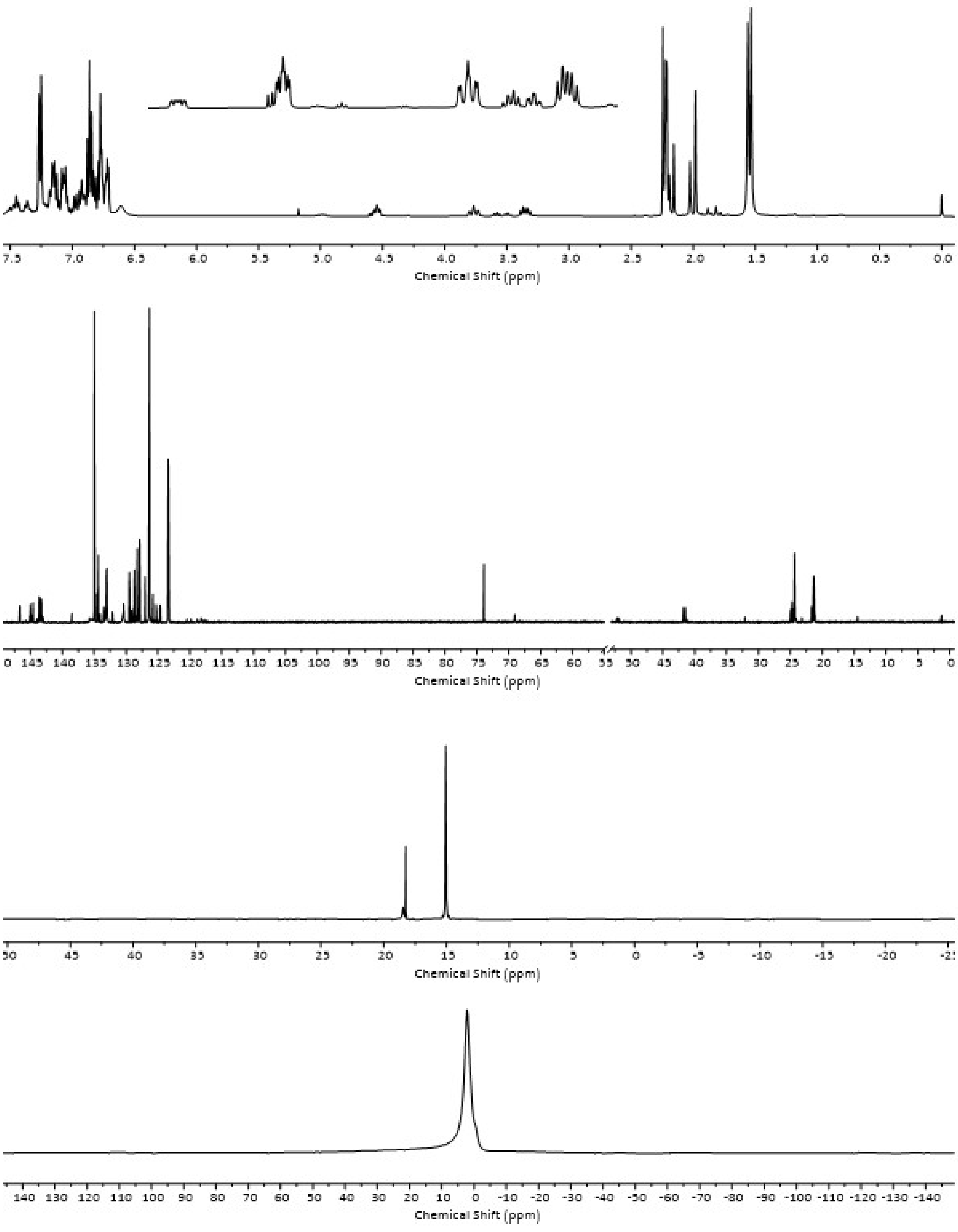

Figure S28: ^{1}H, ^{13}C (solvent peaks omitted for clarity), ^{31}P and ^{11}B (top to bottom) NMR spectroscopic analysis of epoxide activated intermediates present in catalytic cycle when **SO** is used as a substrate with a **P2**/**B1** catalyst system.

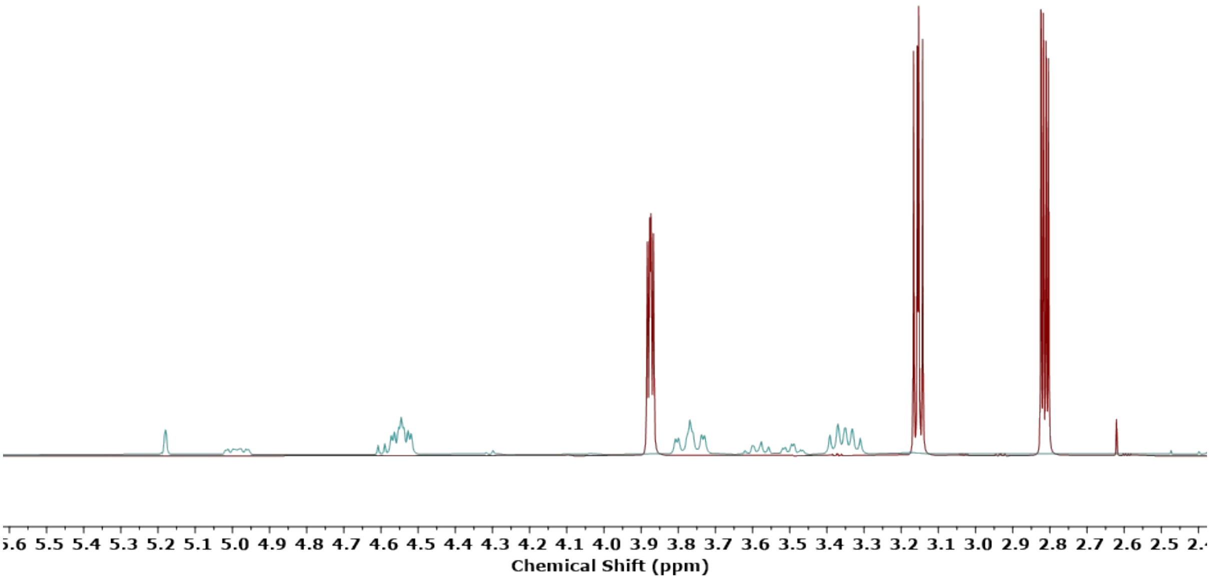

Figure S29: Superimposed spectra showing impact of epoxide activation by P2/B1. Red = Propylene oxide oxide (PO), Blue = SM complex of ring-opened epoxide.

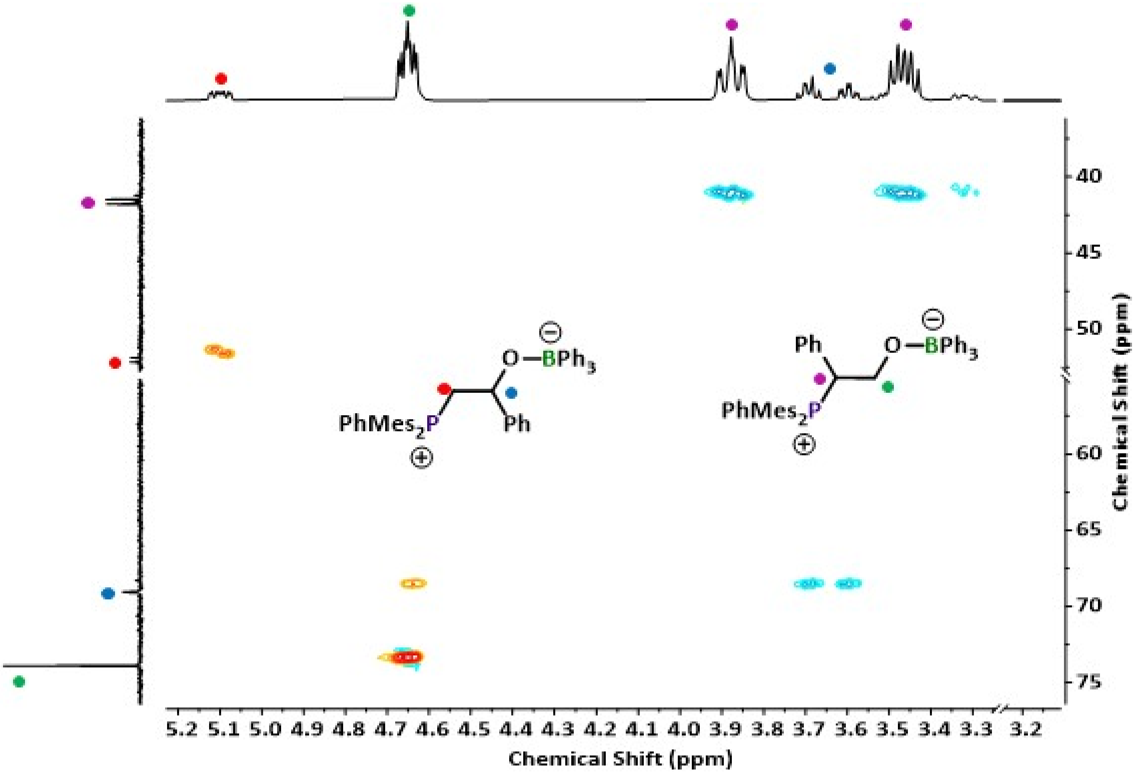

Figure S30: HSQC analysis demonstrating position of LB attack when **SO** is used as a substrate with a **P2**/**B1** catalyst system. Red = CH_2, Blue = CH or CH_3. Using ^{31}P-^{13}C coupling constants, attack of **P2** therefore favours the least hindered carbon, although attack is still possible at the more hindered position. Solvent peaks omitted for clarity.

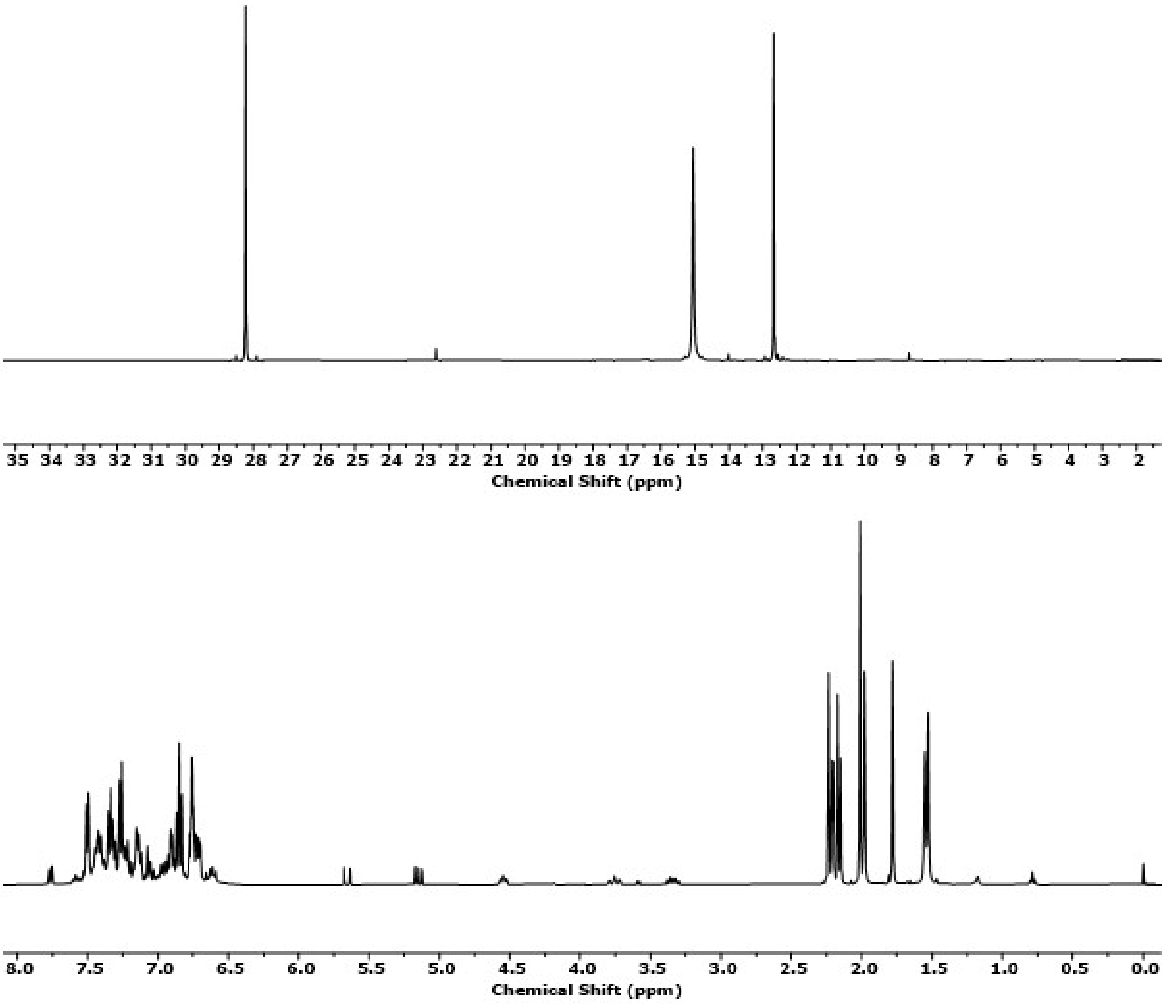

Figure S31: ^{31}P (top) and ^{1}H (bottom) NMR spectra showing heated activated **SO** complex, **$PMes_2Ph.SO.BPh_3$**. Due to multiple positions of phosphine attack, both styrene elimination and other decomposition routes are observed. Since vinyl peaks are not observed during catalysis with **P2/B1**, bulk of the polymer backbone is likely sufficient to prevent attack at the more hindered carbon.

CO_2 Activation and Proposed Mechanism

Since FLPs are known to activate carbon dioxide, a standard experiment was performed to see if CO_2 activation plays a role in the catalysis observed with **P2**/**B1**.

In a glovebox, BPh_3 (0.03 g, 0.12 mmol) and $PMes_2Ph$ (0.043 g, 0.12 mmol) were mixed in toluene-d_8 and transferred to a high-pressure NMR tube. The tube was removed from the glovebox and crude NMR spectra taken to confirm their lack of interaction. The solution was then degassed via successive freeze-pump-thaw cycles and loaded with 2 bar of CO_2, before being left to settle for 3 hours. ^{31}P, ^{11}B and ^{13}C NMR spectra were then taken and the lack of CO_2 activation confirmed since no changes could be observed.

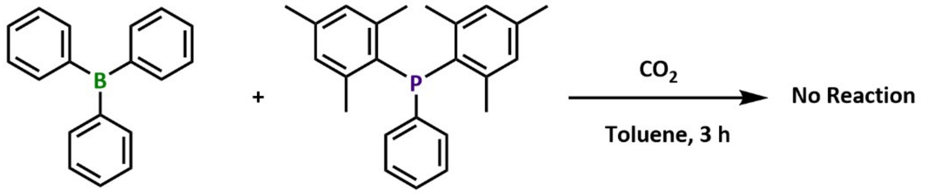

On the basis of this result and the ring-opening events demonstrated above, we propose a reaction mechanism similar to that reported with other aryl borane-containing systems.[7]

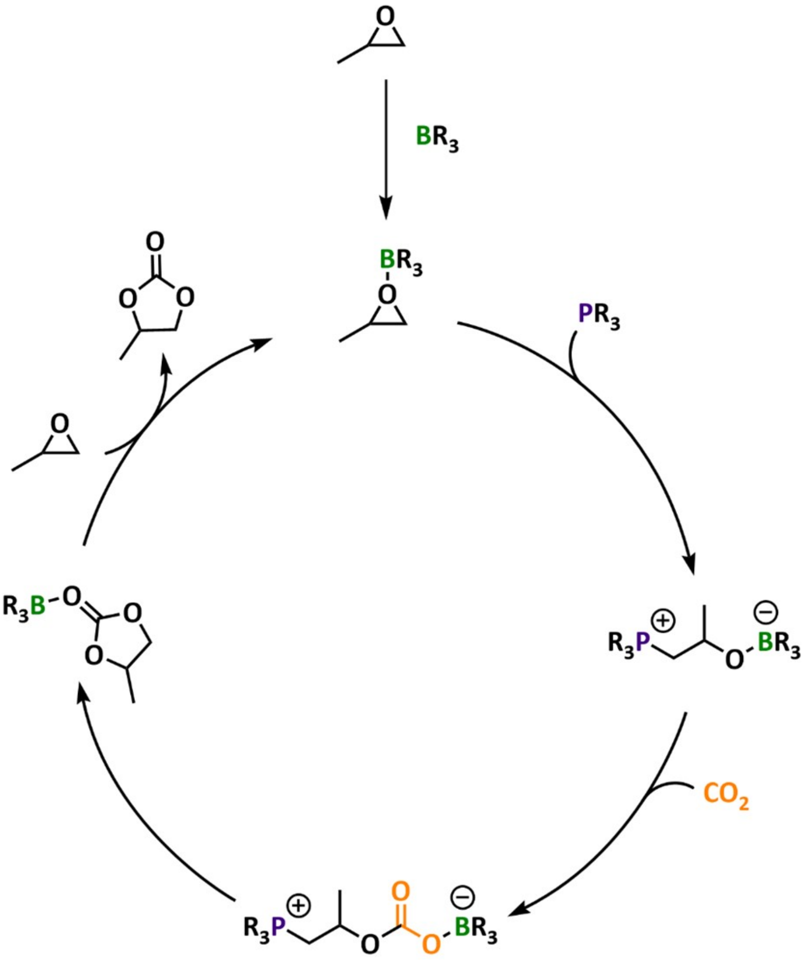

Figure S32: Proposed catalytic cycle using **PO** as a substrate.

5. CO_2 Insertion to Oxetane Substrates

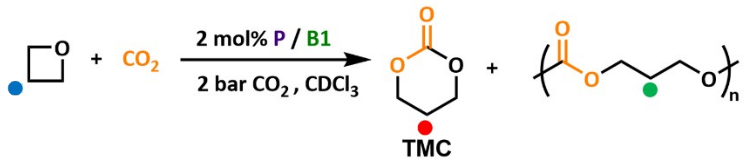

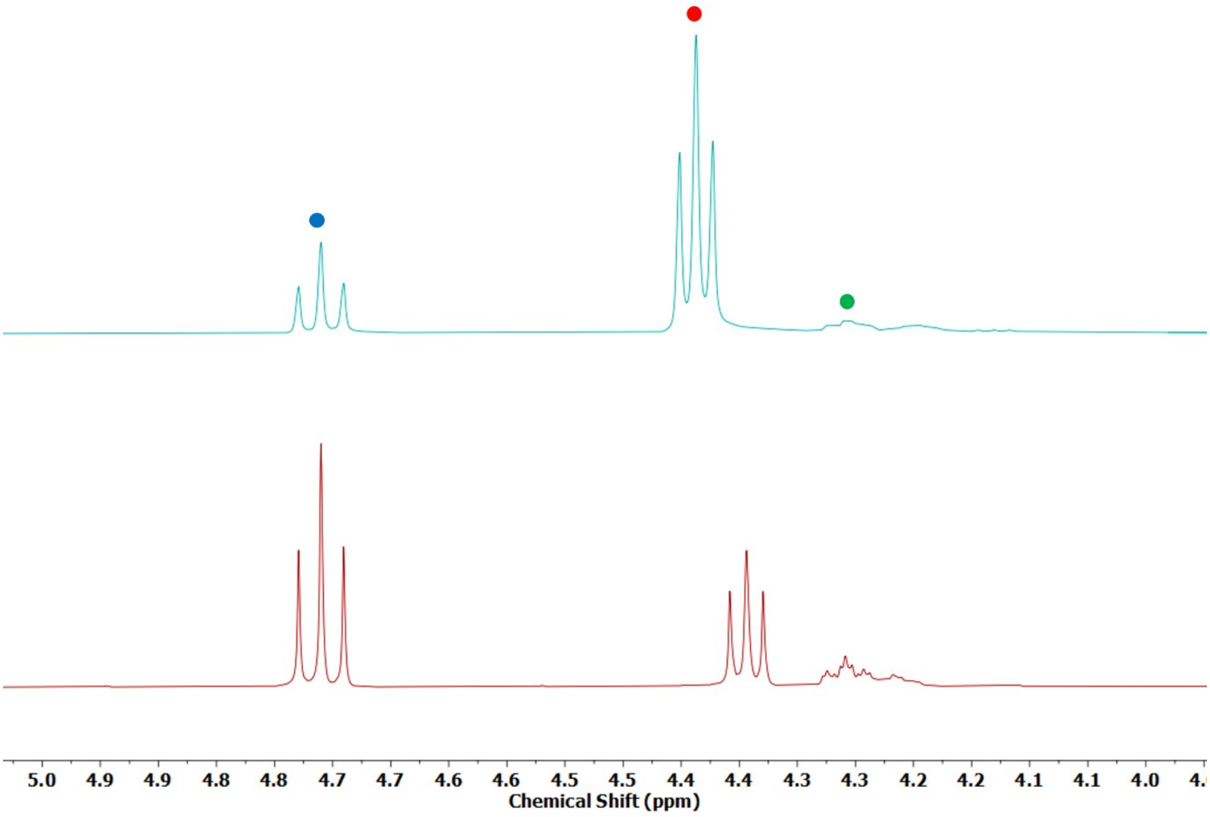

Figure S33: Temperature influence on trimethylene carbonate vs polycarbonate selectivity on addition of CO_2 to oxetane with a **P2**/**B1** catalytic system.

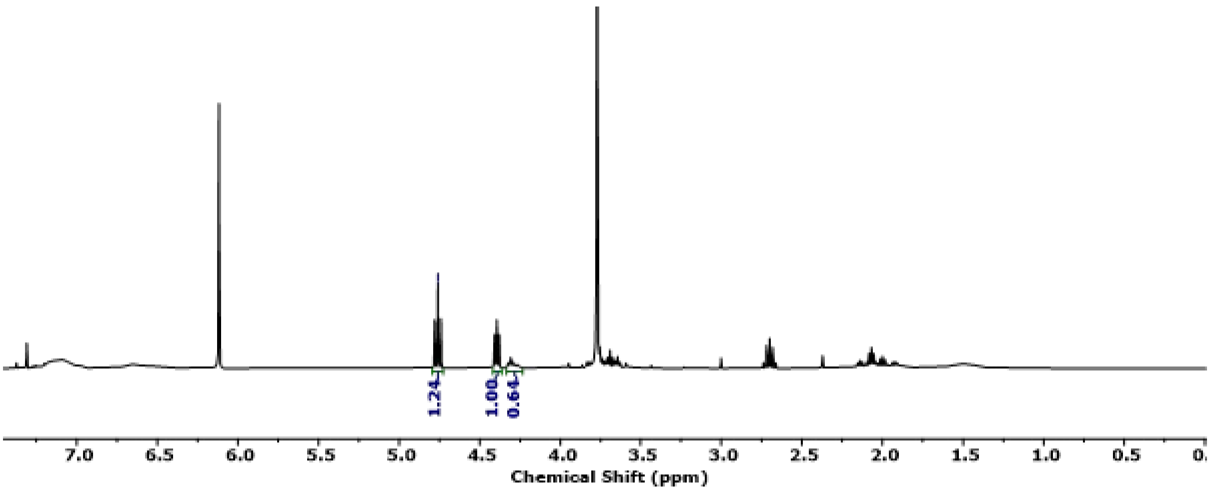

Figure S34: Expanded ^{1}H NMR spectrum of CO_2 addition to oxetane at 120 °C using a **P2**/**B1** catalytic system.

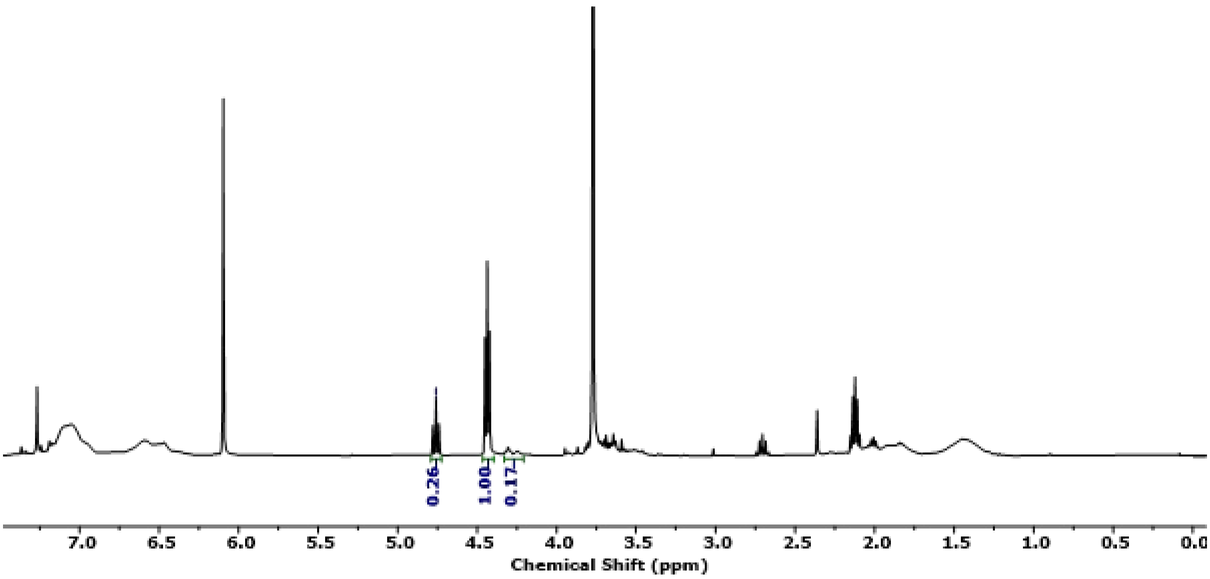

Figure S35: Expanded ^{1}H NMR spectrum of CO_2 addition to oxetane at 100 °C using a **P2**/**B1** catalytic system.

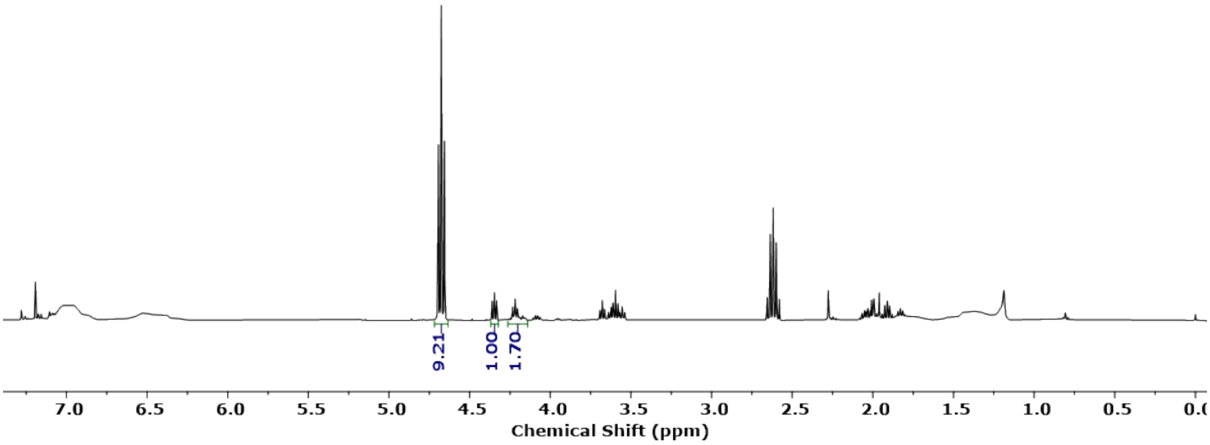

Figure S36: Expanded ^{1}H NMR spectrum of CO_2 addition to oxetane at 120 °C using a **P1**/**B1** catalytic system.

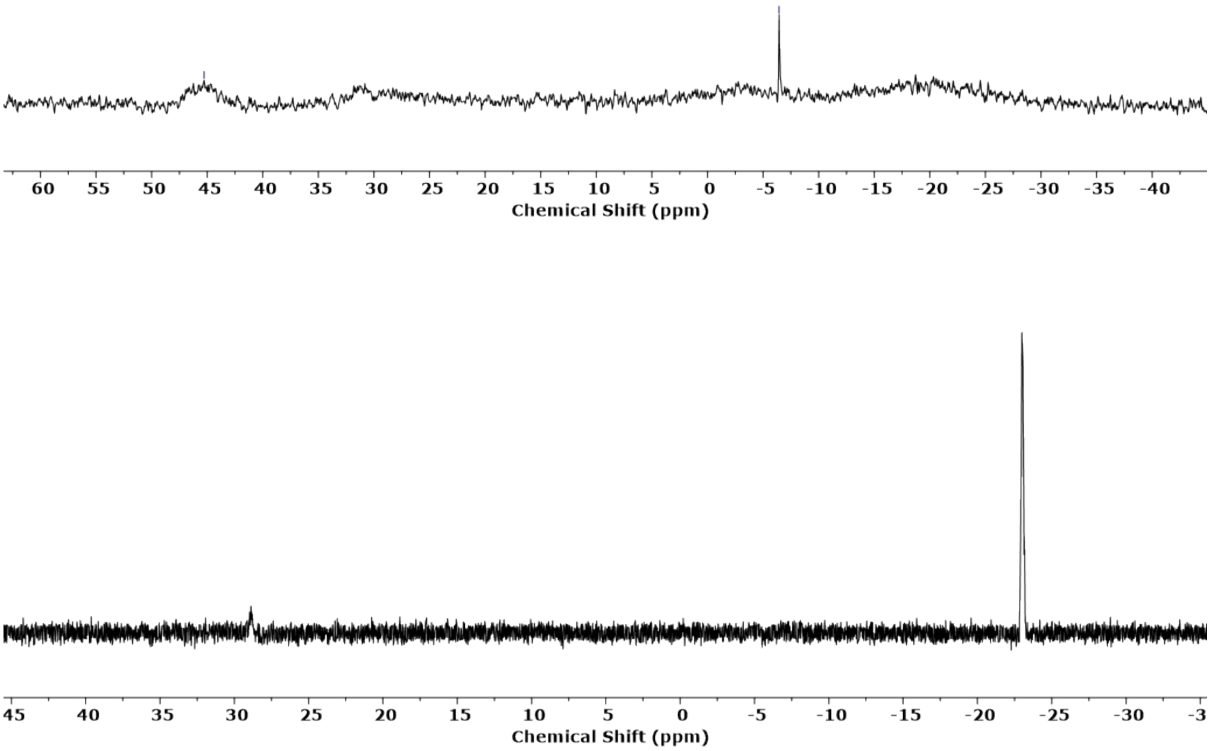

Figure S37: ^{11}B (top) and ^{31}P (bottom) NMR spectra for attempted CO_2 insertion to bromooxetane under reaction conditions shown in Entry 1, table 1.

6. Consecutive Reaction Cycles

General reaction procedure

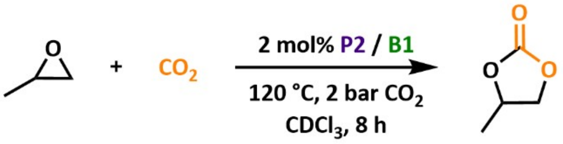

In a glovebox, two separate stock solutions of **P2** (0.039 g, 0.2 mL, 1.8 µmol P moieties) and **B1** (0.034 g, 0.2 mL, 1.8 µmol B moieties) in $CDCl_3$ were combined in an ampoule and stirred to ensure efficient mixing before addition of a 1,3,5-trimethoxybenzene internal standard solution (0.08 g in 0.1 mL $CDCl_3$). Next, a solution of the desired cyclic ether substrate (0.8 mmol, in 0.2 mL $CDCl_3$) was added and a small portion taken and dilute for t_0 analysis by ^{1}H NMR spectroscopy. The reaction vessel was then sealed with a Young's tap and removed from the glovebox before being connected to a Schlenk line equipped with a CO_2 cylinder via a 3-way joint connection. The reaction mixture was degassed via 3 successive freeze-pump-thaw cycles before being allowed to warm to room temperature. Maintaining an inert atmosphere with stirring, CO_2 was added (2 bar) before sealing of the ampoule and placing in an oil bath at 120 °C for 8 hours. After the reaction time, the ampoule was returned to the glovebox and the contents taken for analysis by NMR spectroscopy to determine the obtained conversion. Once analysis was complete, the reaction mixture was precipitated into anhydrous n-hexane and left to settle in the glovebox freezer overnight (-35 °C). The precipitate was subsequently filtered and rinsed with more n-hexane before drying under vacuum. The recovered polymer was then used in the next reaction cycle as described above.

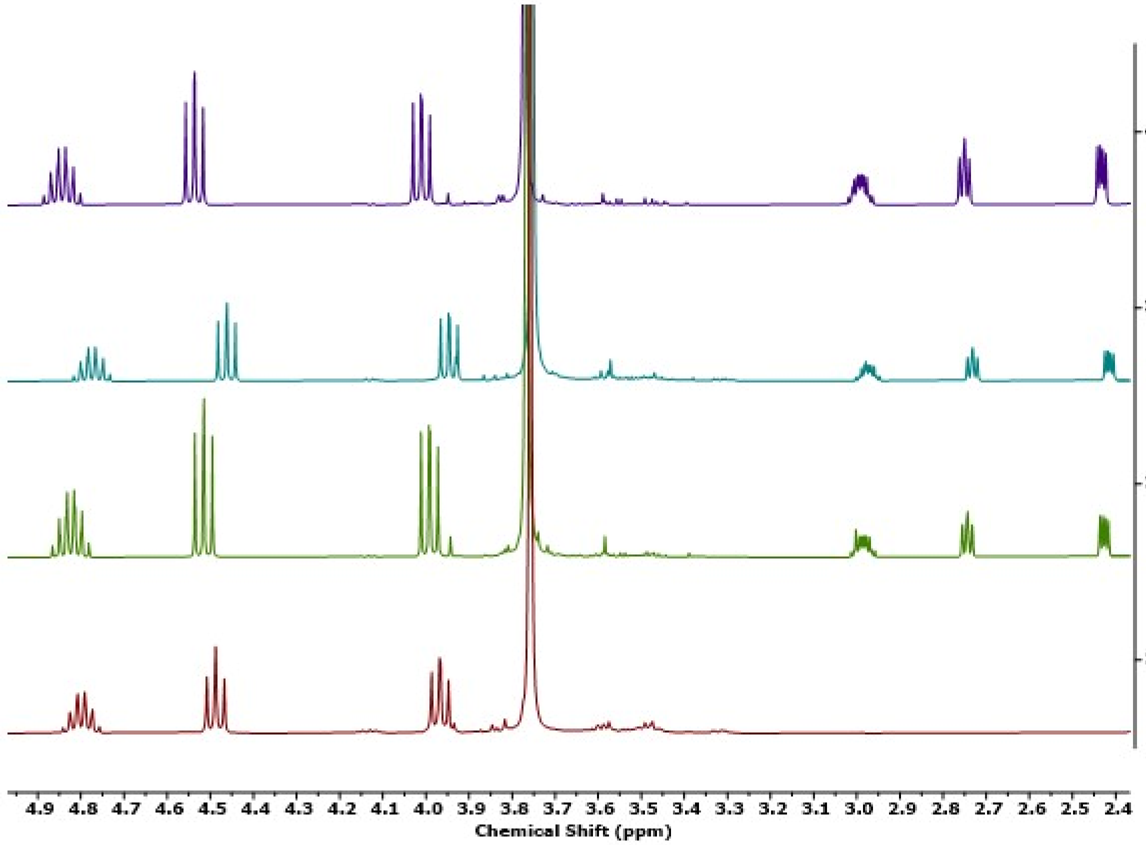

Figure S38: Magnified product/reagent region of ^{1}H NMR spectroscopic analysis of obtained conversions for consecutive reaction cycles 1-4 as numbered on vertical axis.

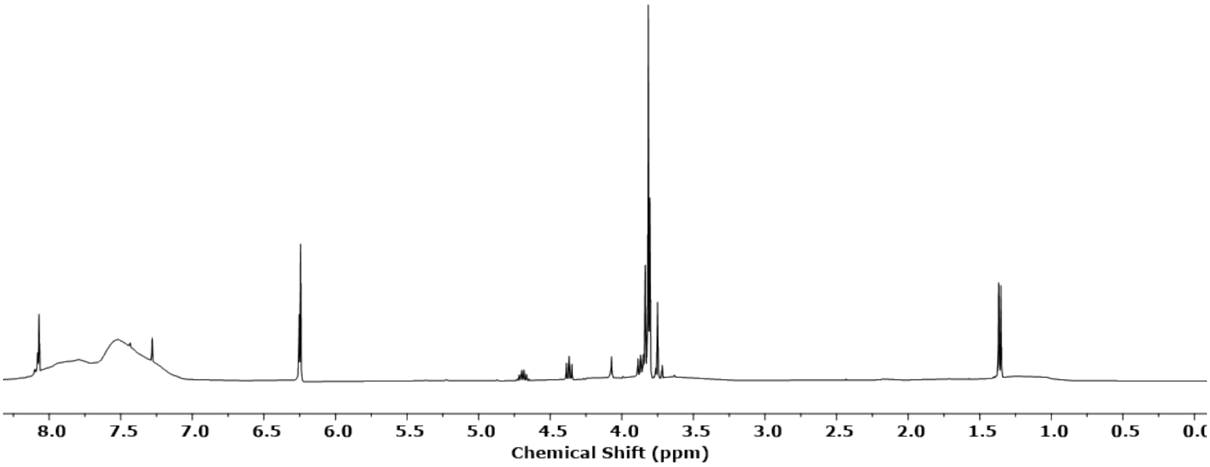

Figure S39: ^{1}H NMR spectrum demonstrating **P2/B1** remain active to achieve >99% conversion over 48 hours for 5th precipitation/recovery cycles.

Standard reactions were also performed using polymeric catalysts, employing fresh addition of substrate as opposed to catalyst recovery and subsequent reuse.

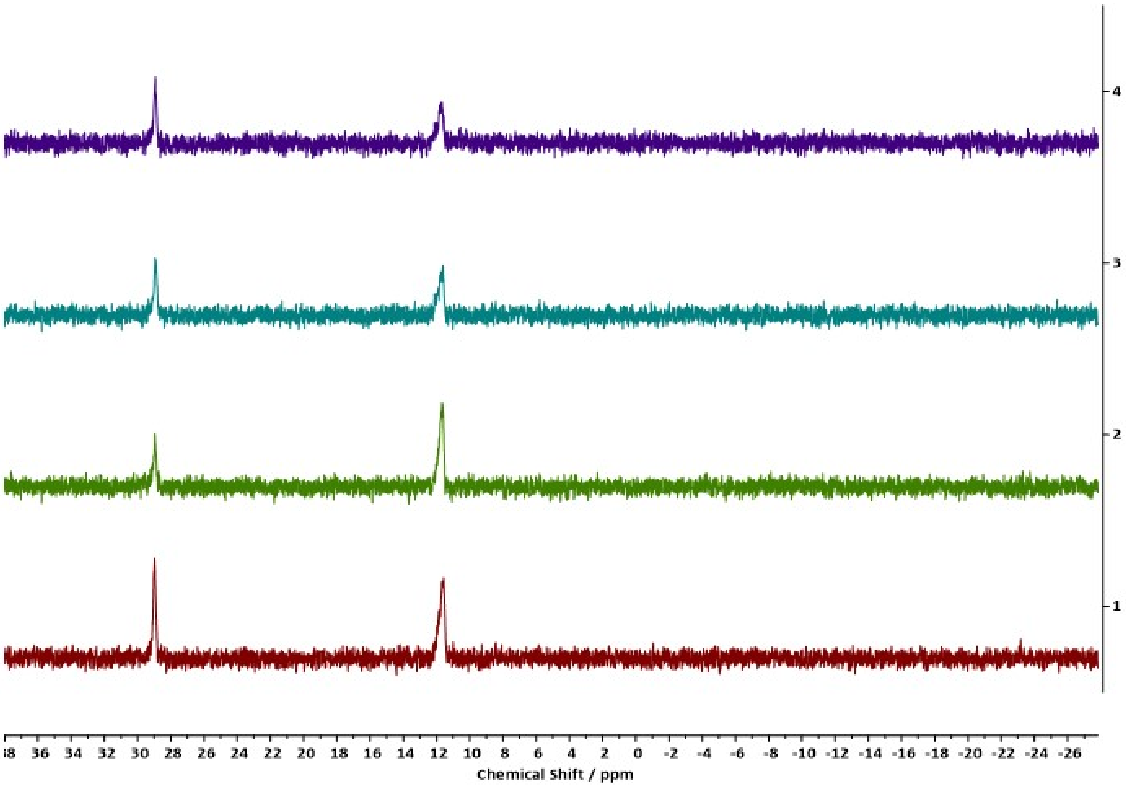

Figure S40: ^{31}P NMR spectra for reaction cycles 1-4 as numbered on vertical axis. Phosphine oxidation can be observed however there is little subsequent increase across cycles.

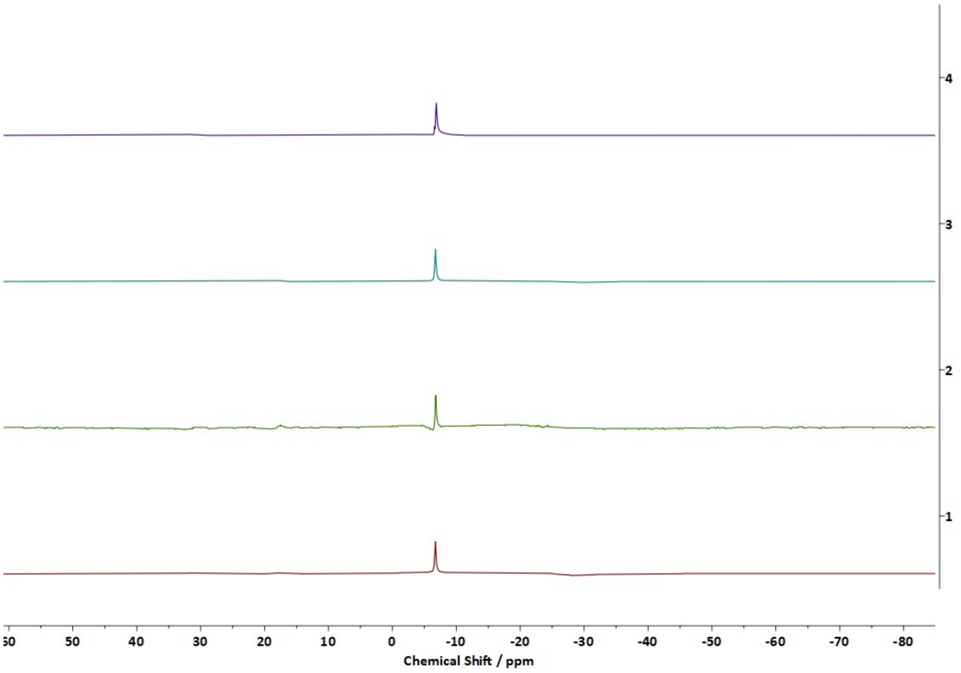

Figure S41: ^{11}B NMR spectra for reaction cycles 1-4 as numbered on vertical axis. A principle peak associated with a 4-membered boron-containing species can be observed across all cycles with no evident decomposition.

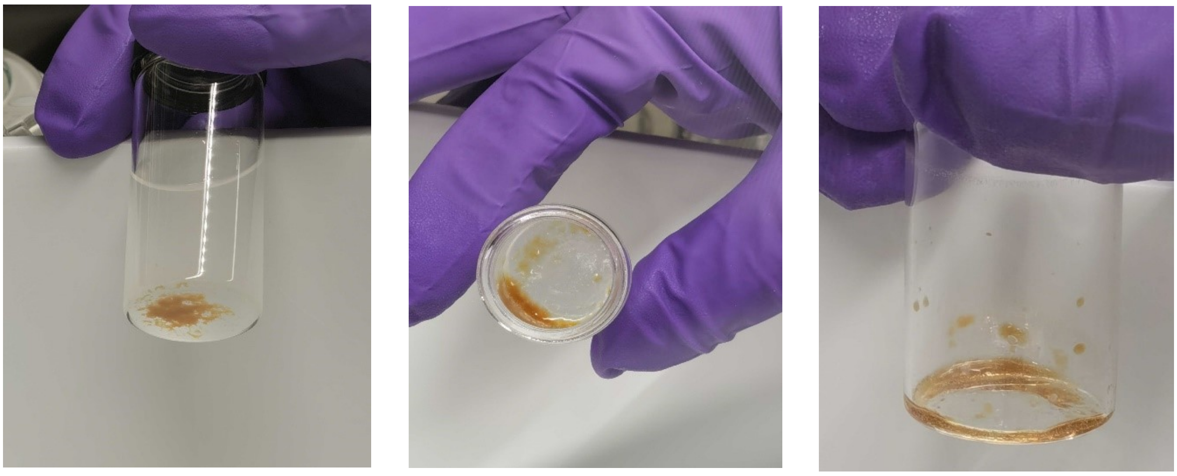

Figure S42: Images showing precipitated copolymer catalyst after reaciton cycle 2 (left) and swelling of recovered copolymer catalysts upon addition of solvent and fresh substrate for new reaction cycle (centre and right).

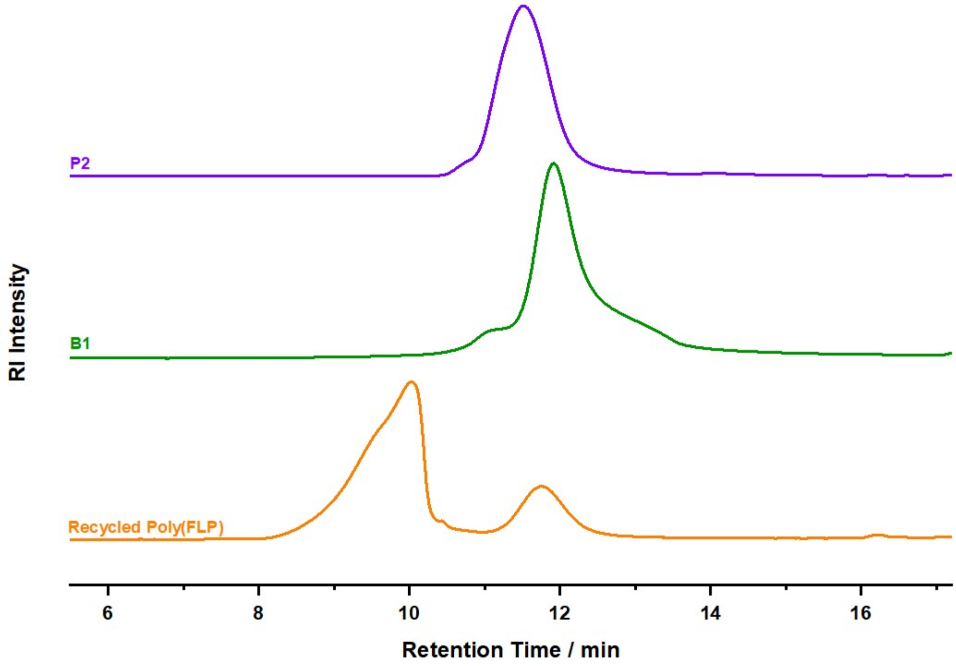

Figure S43: GPC trace showing increase in molecular weight of recovered copolymers after 4 reaction cycles (bottom) as a result of crosslinking of original copolymer catalysts (top and middle).

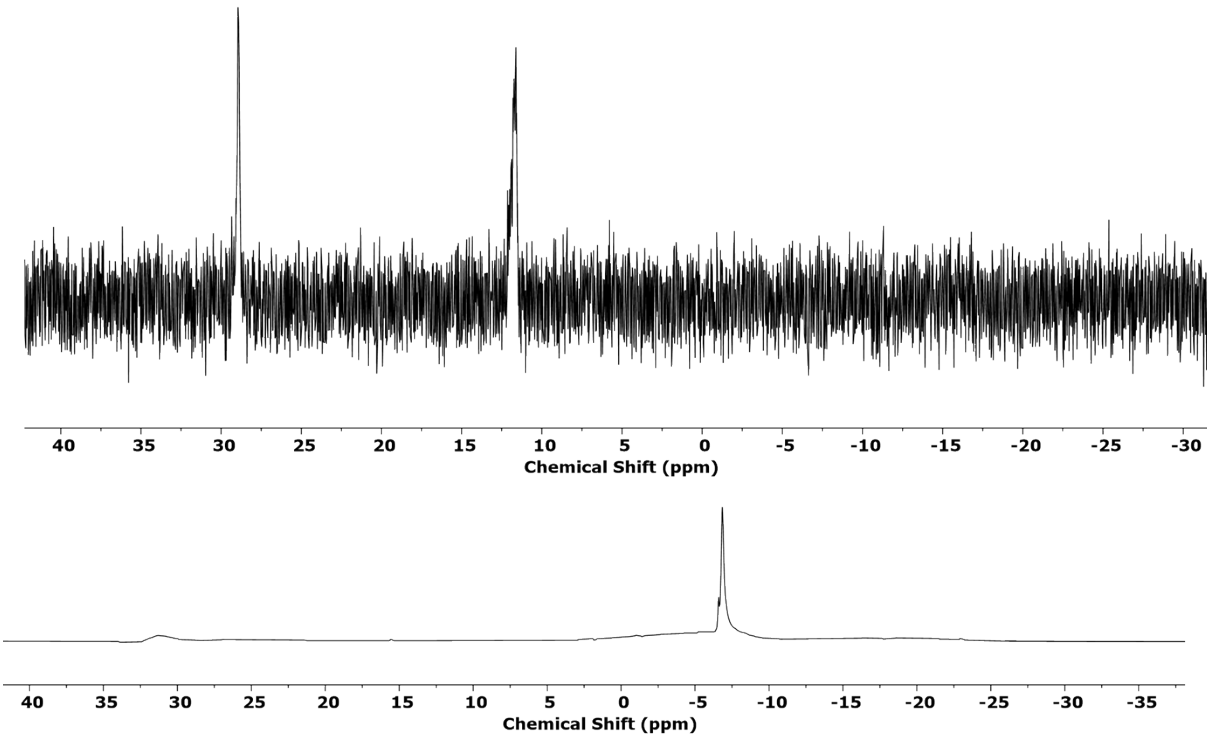

Figure S44: ^{31}P (top) and ^{11}B (bottom) NMR spectra of recovered polymer catalyst after 4 reaction cycles.

7. References

1 M. Wang, F. Nudelman, R. R. Matthes and M. P. Shaver, *J. Am. Chem. Soc.*, 2017, **139**, 14232–14236.

2 U. Yolsal, M. Wang, J. R. Royer and M. P. Shaver, *Macromolecules*, 2019, **52**, 3417–3425.

3 J. A. Buonomo, C. G. Eiden and C. C. Aldrich, *Synth.*, 2018, **50**, 278–281.

4 U. Yolsal, T. A. R. Horton, M. Wang and M. P. Shaver, *J. Am. Chem. Soc.*, 2021, **143**, 12980–12984.

5 M. Wang, J. Holland, T. A. R. Horton, U. Yolsal and M. P. Shaver, *Polymer*, 2022, 124576.

6 J. A. Buonomo, C. G. Eiden and C. C. Aldrich, *Chem. - A Eur. J.*, 2017, **23**, 14434–14438.

7 K. A. Andrea and F. M. Kerton, *ACS Catal.*, 2019, **9**, 1799–1809.
